# Tribranched Multiblock Copolymers Mimicking the Molecular Claw Design in N‑Type Conjugated Polymers for High-Yield Semiconducting Carbon Nanotube Sorting and Phototransistor Memory Applications

**DOI:** 10.1021/acsami.6c08012

**Published:** 2026-06-12

**Authors:** Yu-Che Kan, Shuto Yamamoto, Yu-Chun Huang, Yi-Hsuan Tung, Guo-Hao Jiang, Ming-Han Chen, Chien-Chung Shih, He Sun, Tomoya Higashihara, Yan-Cheng Lin

**Affiliations:** † Department of Chemical Engineering, 34912 National Cheng Kung University, Tainan 70101, Taiwan; ‡ Department of Organic Materials Science, Graduate School of Organic Materials Science, Yamagata University, 4-3-16 Jonan, Yonezawa, Yamagata 992-8510, Japan; § Department of Chemical Engineering and Materials Engineering, 34883 National Yunlin University of Science and Technology, Yunlin 64002, Taiwan; ∥ Advanced Research Center for Green Materials Science and Technology, National Taiwan University, Taipei 10617, Taiwan; ⊥ Program on Smart and Sustainable Manufacturing, Academy of Innovative Semiconductor and Sustainable Manufacturing, National Cheng Kung University, Tainan 70101, Taiwan

**Keywords:** naphthalene diimide, conjugated polymers, block copolymers, single-walled carbon nanotubes, photomemory

## Abstract

N-type semiconducting polymers are crucial for complementary logic circuits but typically suffer from strong self-aggregation and electronic instability. To address these challenges, a molecular claw architecture for n-type semiconducting tribranched multiblock copolymers is proposed. Two polymers, tAB-1 and tAB-2, are synthesized by incorporating polyisobutylene soft segments into the backbone of a rigid naphthalene-diimide-based semiconducting polymer. The high ratio of soft segments in tAB-2 disrupts backbone planarity and effectively reduces self-aggregation. Consequently, the tAB-2 polymer achieves a sorting purity exceeding 99.9%. Although the polymers have a hyperbranched rather than a well-defined three-arm architecture, this molecular claw-like topology increases the contact area with the nanotube surface. It facilitates the capture of small-diameter carbon nanotubes that possess high intrinsic defect densities. Structural analysis reveals that the composite forms debundled fibers with an increased paracrystalline disorder within the polymer stacks. This loose molecular packing generates abundant charge-trapping sites at the polymer–nanotube heterointerface. Consequently, the tAB-2 phototransistor memory exhibits exceptional electrical characteristics. It achieves a wide memory window of 80 V and a high memory ratio of 10^5^. Furthermore, the device demonstrates stable multibit and dynamic operation at an ultralow drain voltage of −10 mV, with no gate bias, to minimize power consumption. This study confirms that topological engineering effectively balances sorting purity with defect engineering for high-performance organic optoelectronic memory.

## Graphical abstract

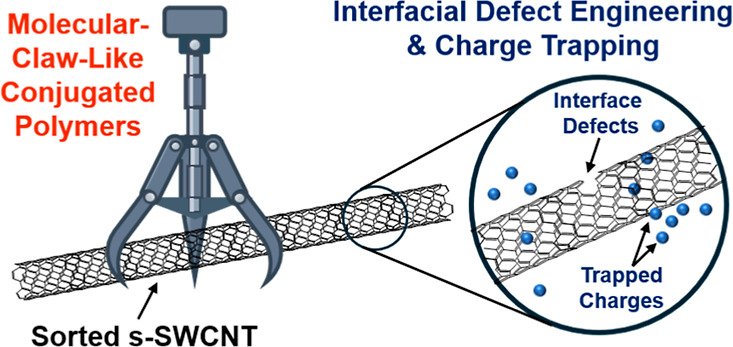

## Introduction

The continuous miniaturization of silicon-based electronics is approaching its physical limits, challenging the sustainability of Moore’s Law.^1^ To overcome these bottlenecks and suppress short-channel effects in ultrascaled devices, material development has shifted toward low-dimensional nanomaterials.^2,3^ Among these candidates, single-walled carbon nanotubes (SWCNTs) stand out due to their unique one-dimensional structure. Since the discovery of SWCNTs by Iijima in 1991,^4^ researchers have prioritized these nanomaterials for next-generation electronics. SWCNTs exhibit exceptional charge carrier mobility^5^ and high mechanical flexibility.^6^ These properties make them ideal candidates for flexible optoelectronic devices and logic circuits. However, standard synthesis methods produce a mixture of metallic and semiconducting species.^7^ Metallic nanotubes create leakage paths in field-effect transistors (FETs), leading to high off-currents, which degrades the device’s on/off current ratio.^8^ To build reliable electronic circuits, scientists have developed several methods for purifying semiconducting SWCNTs (s-SWCNTs). Standard techniques include density gradient ultracentrifugation (DGU),^7^ gel chromatography,^7,9^ and DNA wrapping.^10^ While DGU and gel chromatography are effective, they often require complex procedures or expensive consumables.^11^ DNA wrapping method provides high resolution but suffers from the high costs of the expensive oligonucleotides required.^12^ In contrast, conjugated polymer wrapping (CPW) offers significant advantages, such as high purity, simple operation, and industrial scalability.^13^ It uses selective π–π interactions between polymer backbones and nanotube surfaces to isolate specific s-SWCNTs from the raw mixture.^14^

Early CPW studies utilized homopolymers. Nish et al. used polyfluorene (PFO) derivatives to isolate specific nanotube chiralities.^15^ Other groups investigated polythiophene derivatives such as poly­(3-hexylthiophene-2,5-diyl) (P3HT)^16^ and poly­(3-dodecylthiophene-2,5-diyl) (P3DDT).^17^ These p-type polymers effectively select s-SWCNTs. However, most p-type homopolymers have wide band gaps exceeding 2 eV, which creates a significant energy level mismatch with s-SWCNTs.^18^ Such a misalignment causes an inefficient carrier injection and undesired charge trapping. To address this, scientists introduced donor–acceptor (D–A) copolymers, where the energy levels can be adjusted through molecular engineering.^19,20^ This tunability enhances the interaction between polymers and SWCNTs and improves the charge transport.^21,22^ Despite these advances, n-type sorting polymers remain underdeveloped compared to p-type materials. Most existing sorting polymers act as a hole transport medium in the polymer/s-SWCNT composite channel.^15,17^ N-type polymers offer a necessary alternative for complementary circuits. Naphthalene diimide (NDI)-based polymers are up-and-coming candidates due to their electron-deficient nature.^23,24^ They possess deep LUMO levels that align well with the conduction band of s-SWCNTs, which promotes efficient electron transport. To expand the library of n-type sorting systems beyond conventional NDI frameworks, recent research has actively explored alternative electron-deficient architectures, including perylene diimide (PDI)-based polymers with extended pi-conjugation that can be utilized as molecular tweezers,^25^ benzothiadiazole-based copolymers that use the poor coplanarity backbone to achieve excellent selectivity of s-SWCNT and chirality,^26^ and all-acceptor polymers engineered for lowering the LUMO to match the SWCNT energy level.^21^ However, these emerging n-type sorting polymers often exhibit low dispersion yields due to weak backbone interactions or strong self-aggregation.^26^ Furthermore, electronic devices utilizing these polymers frequently suffer from severe hysteresis originating from charge trapping at the polymer–nanotube interface.^18^ In addition, the insulating polymer residues are difficult to remove completely, where these residues may increase tunneling barriers and hinder carrier transport between nanotubes.^18^ Researchers are still struggling to balance purity, yield, and electronic stability in n-type polymers within sorting systems. Furthermore, integrating sorted s-SWCNTs into nonvolatile memory warrants further exploration. The rapid growth of data-intensive computing has exposed the limitations of the traditional von Neumann architecture. The physical separation of processing and storage creates a bottleneck known as the memory wall.^27,28^ Phototransistor memory offers a viable solution to this problem. Unlike two-terminal memristors, which often suffer from signal crosstalk, three-terminal phototransistors provide signal amplification and nondestructive readout. Device performance depends heavily on the charge-trapping mechanism at the interface.^29^ Previous studies integrated s-SWCNT channels with polymer electrets or floating-gate architectures to store charge.^30^ However, the influence of polymer topology on defect engineering remains unclear, particularly in conjugated block copolymer systems.

To unveil this interplay, a new molecular design strategy is required to enhance both sorting efficiency and memory performance. Building on our previous success in developing linear ABA-type triblock copolymers^31^ and (AB)_*n*_ multiblock copolymers^32^ composed of rigid naphthalene–diimide–bithiophene-based semiconducting polymer (PNDI2T) and soft polyisobutylene (PIB) segments, which effectively mitigated the trade-off between charge carrier mobility and stretchability. The general framework of utilizing nonconjugated soft segments within block copolymer architectures has recently demonstrated profound efficacy in s-SWCNT sorting systems. Recent studies demonstrate that introducing flexible, nonconjugated blocks effectively disrupts the intrinsic self-aggregation and π–π stacking of rigid backbones, promoting selective wrapping around individual carbon nanotubes.^33^ Furthermore, these flexible segments provide interfacial elasticity, allowing the polymer chains to conform to the specific surface curvature of s-SWCNTs adaptively.^34^ This curvature-matching mechanism efficiently excludes metallic species, yielding purities exceeding 99%. Crucially, the highly solvated soft blocks extend outward to form a robust steric barrier, thereby suppressing secondary bundling of polymer-SWCNT complexes and ensuring excellent long-term solution stability.^35^ Inspired by these multifaceted benefits, we extend this structural paradigm to a molecular claw architecture. In practice, by incorporating 3-arm star-branched PIB segments into the PNDI2T backbone, tribranched multiblock copolymers with a unique topology are created. Drawing inspiration from the molecular claw concept, which utilizes cavity-like structures to capture specific guest molecules,^36^ tribranched multiblock copolymers were designed to selectively grasp s-SWCNTs. Specifically, two such polymers, designated tAB-1 and tAB-2, were synthesized, containing 11 and 34 wt % soft PIB segments, respectively. These materials are compared against a rigid control homopolymer, PNDI2T. The sorting performance was rigorously evaluated using ultraviolet–visible–near-infrared (UV–vis–NIR) absorption, Raman spectroscopy, and photoluminescence excitation (PLE) mapping. Experimental results indicate that the tribranched architecture significantly improves sorting purity compared to the linear control. Raman and PLE analyses confirm that tAB-2 preferentially selects s-SWCNTs with smaller diameters. Due to the higher surface curvature of these smaller diameter nanotubes, the tAB-2/s-SWCNTs have more defects than the homopolymer ones. This is conducive to creating a heterojunction at the interface between the electron-deficient n-type polymer and the p-type s-SWCNTs. This heterojunction facilitates efficient charge transfer and trapping, which is essential for the stable operation of the memory device. Consequently, phototransistor memory devices fabricated using tAB-2 sorted inks achieved a wide operation window (Δ*V*_TH_) of 80 V and a high memory ratio of 10^5^. This work establishes a design guideline for tribranched multiblock copolymers, demonstrating how topologies and architectures can optimize the trade-off between purity and defect engineering for high-performance optoelectronic memory.

## Results and Discussion

### Synthesis and Characterization of Tribranched Multiblock Copolymers

We previously synthesized linear (AB)_*n*_ multiblock copolymers (A = PNDI2T and B = PIB) via statistical ternary copolymerization based on the Migita–Kosugi–Stille coupling reaction among 5,5′-bis­(trimethylstannyl)-2,2′-bithiophene (2T), 4,9-dibromo-2,7-bis­(2-decyltetradecyl)­benzo­[lmn]­[3,8]­phenanthroline-1,3,6,8­(2*H*,7*H*)-tetraone (Br-NDI-Br) and α,ω-chain-end-functionalized PIB bearing 5-bromothien-2-yl groups (Br-PIB-Br).^32^ In this study, novel tribranched multiblock copolymers, tAB-1 and tAB-2, were synthesized by modifying the above protocol, in which Br-PIB-Br was replaced with a three-arm star-branched PIB bearing a 5-bromothien-2-yl group at the terminus of each arm (tPIB-Br) (Scheme 1). The precursor tPIB-Br was synthesized via living cationic polymerization of isobutene using a trifunctional core initiator, followed by in situ quenching with an excess of thiophene. Subsequent bromination at the 5-position of the thiophene units introduced at the chain ends afforded tPIB-Br (Scheme S1). tPIB-Br possessed a number-average molar-mass (*M*_n_) value of 8600 g/mol and a molar-mass dispersity (*D̵*_M_) value of 1.09, as confirmed by SEC (Figure S1). The quantitative introduction of the 5-bromothien-2-yl group was confirmed by ^1^H NMR (Figure S2). The two polymers, tAB-1 (PIB: 11 wt %) and tAB-2 (PIB: 34 wt %), were synthesized via the statistical terpolymerization between A_2_ (2T), B_2_ (Br-NDI-Br), and B′_3_ (tPIB-Br) type comonomers based on the Migita–Kosugi–Stille coupling reaction in the presence of Pd_2_(dba)_3_ and P­(*o*-tol)_3_ in toluene at the reflux temperature (Scheme 1). In the initial polymerization step, Br-NDI-Br and 2T were employed in nearly equimolar amounts to promote the formation of high-molecular-weight PNDI2T segments, while a slight excess of 2T was used to suppress residual bromine end groups. After 1 h of polymerization, aliquot SEC measurements confirmed the formation of the PNDI2T first block, after which tPIB-Br was introduced to construct the tribranched multiblock architecture. Since tPIB-Br acts as a trifunctional macromonomer, the feed ratios were carefully adjusted so that the total numbers of stannyl and bromo functionalities remained nearly equimolar in order to avoid excessive cross-linking and gelation. Furthermore, polymerization was initially investigated under low PIB-content conditions to suppress possible network formation arising from the tribranched PIB units. Therefore, tAB-1 was designed as a low-PIB-content polymer, whereas the PIB content in tAB-2 was increased by increasing the feed ratio of tPIB-Br. More detailed synthetic procedures were provided in the Experimental Section (Supporting Information). The synthetic results are summarized in Table 1. The *M*_n_ values for tAB-1 and tAB-2 were 35,700 g/mol and 36,000 g/mol, respectively, as determined by SEC (Figures S4 and S5). Indeed, a clear shift in the top peak to a higher molecular weight region is observed in the SEC UV traces for tAB-1 and tAB-2 compared to those for the precursor first block of PNDI2T (*M*_n_ = 20,000 g/mol and *D̵*_M_ = 2.09 for tAB-1 and *M*_n_ = 11,100 g/mol and *D̵*_M_ = 1.73 for tAB-2) and tPIB-Br (*M*_n_ = 8600 g/mol), indicating a high blocking efficiency. The weight compositions of the PNDI2T and PIB segments were determined by ^1^H NMR to be 89:11 (tAB-1) and 66:34 (tAB-2) based on the intensity ratio between specific signals of four *N*-methylene protons ***a*** and those of six methyl protons ***g*** assigned to the monomer repeating units of PNDI2T and PIB segments, respectively (Figures S6 and S7).

**Scheme 1:**
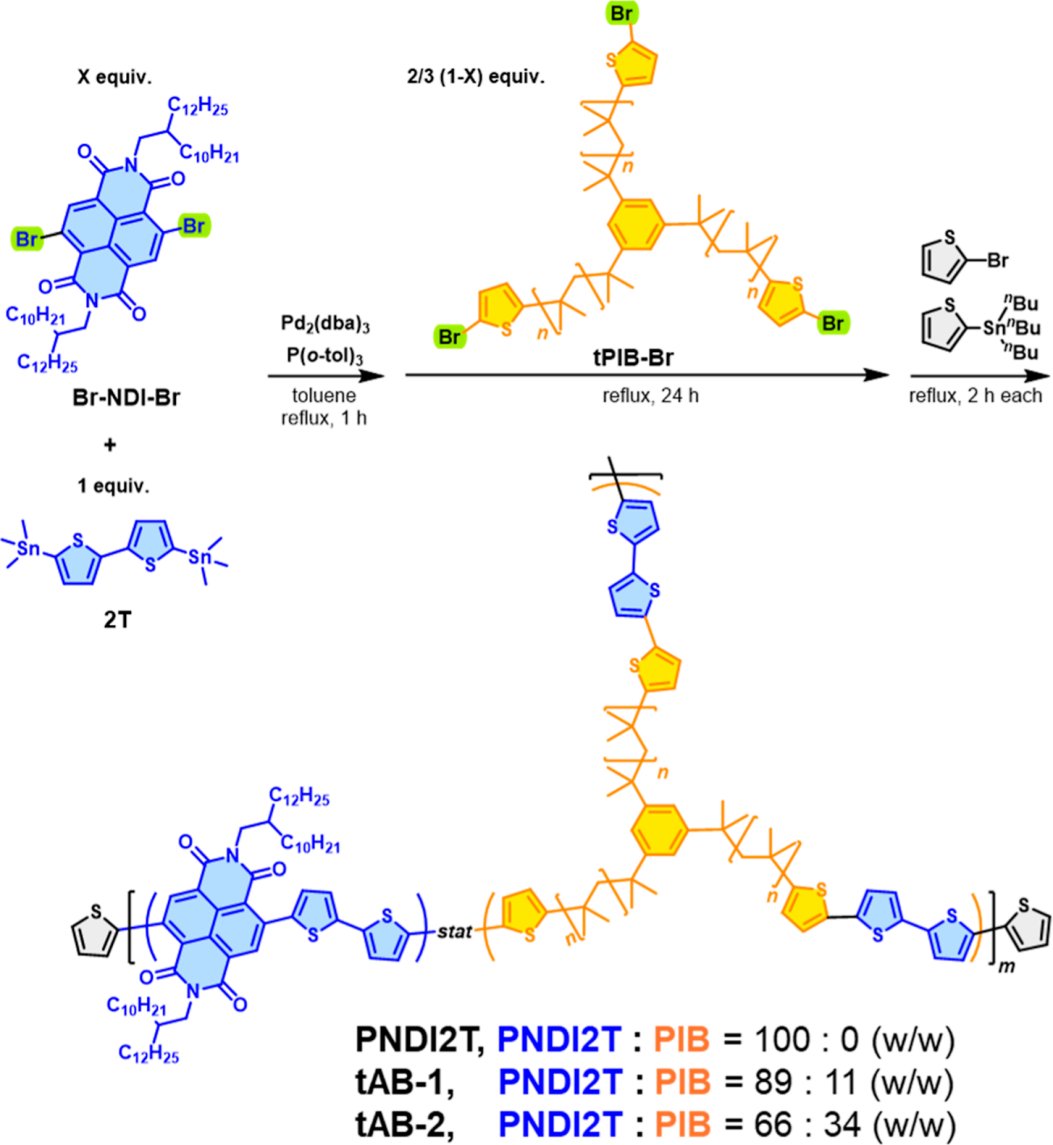
Synthesis of the Tri-Branched Multiblock Copolymers

**Table 1:** Synthetic Results of the Studied Polymers, Including the Molecular Weights and Compositions

	PNDI2T		tPIB-Br		tribranched multiblock copolymers		
polymer	*M*_n_ ^a^	*D̵*_M_ ^a^	*M*_n_ ^a^	*D̵*_M_ ^a^	*M*_n_ ^a^	*D̵*_M_ ^a^	composition (PNDI2T/PIB)^b^
PNDI2T	66,200	1.69	N/A	N/A	N/A	N/A	100:0
tAB-1	20,000	2.08	8600	1.09	35,700	3.51	89:11
tAB-2	11,000	1.73	8600	1.09	36,400	2.98	66:34

a Determined by SEC in chloroform at 40 °C based on a calibration using polystyrene standards.

b Determined by ^1^H NMR in C_2_D_2_Cl_4_ at 100 °C based on the signal intensity ratio for each block segment.

The thermal properties of the studied polymers were evaluated by thermogravimetric analysis (TGA). Samples were heated under a nitrogen atmosphere at a rate of 10 °C/min, and the 5 wt % weight-loss temperatures (*T*_d_^5%^) were determined. The TGA curves are shown in Figure S8. All copolymers exhibited *T*_d_^5%^ over 400 °C, indicating high thermal stability (*T*_d_^5%^ = 412 °C for tAB-1 and *T*_d_^5%^ = 400 °C for tAB-2) (Table S1). Although a slight decrease in thermal stability was observed as PIB content increased, the materials maintained excellent thermal robustness. This behavior is attributed to the high crystallinity of PNDI2T and the intrinsic thermal stability of the PIB segments, which are composed of saturated hydrocarbons. Next, the thermal phase-transition behavior of the studied polymers was investigated by differential scanning calorimetry (DSC) (Figure S9 and summarized in Table S1). In all cases, no glass transition temperature (*T*_g_) attributable to the tri-PIB segment was observed. This result suggests that the crystallinity of the rigid semiconducting PNDI2T backbone was preserved even after the introduction of the three-armed star-branched PIB segments. Instead, a melting temperature (*T*_m_) derived from the PIB chains was observed at −40 °C for tAB-1, while the *T*_m_ and crystallization temperature (*T*_c_) associated with the PNDI2T chains were observed at 240 and 230 °C, respectively. Similarly, the *T*_m_ of the PIB chains appeared at −27 °C for tAB-2, whereas the *T*_m_ and *T*_c_ of the PNDI2T chains were detected at 259 and 247 °C, respectively. The presence of multiple thermal transitions associated with each polymer segment suggests the formation of a certain degree of phase-separated domains between the PNDI2T and PIB segments.

These multiblock copolymers are designed to investigate conformational effects during the s-SWCNT sorting process. Figure 1a shows the normalized optical absorption spectra. In toluene, tAB-2 exhibits a blue-shifted peak, whereas tAB-1 and PNDI2T show a smaller shift. This trend indicates that the soft segments disrupt backbone planarity and ordered aggregation. Following the methods by Steyrleuthner et al. and Wang et al., we calculate the aggregation fraction (*f*_aggregated_) to confirm these characteristics.^37,38^ The aggregate spectrum is obtained by subtracting the disordered spectrum in 1-chloronaphthalene from the solution spectrum in toluene. Figure 1b–d demonstrate the polymer aggregation behavior. Among the samples, tAB-2 shows the lowest *f*_aggregated_ value of 23.2%. The reduced self-aggregation in tAB-2 improves its solubility, facilitating dispersion and contributing to a higher sorting yield.^39^

**Figure 1:**
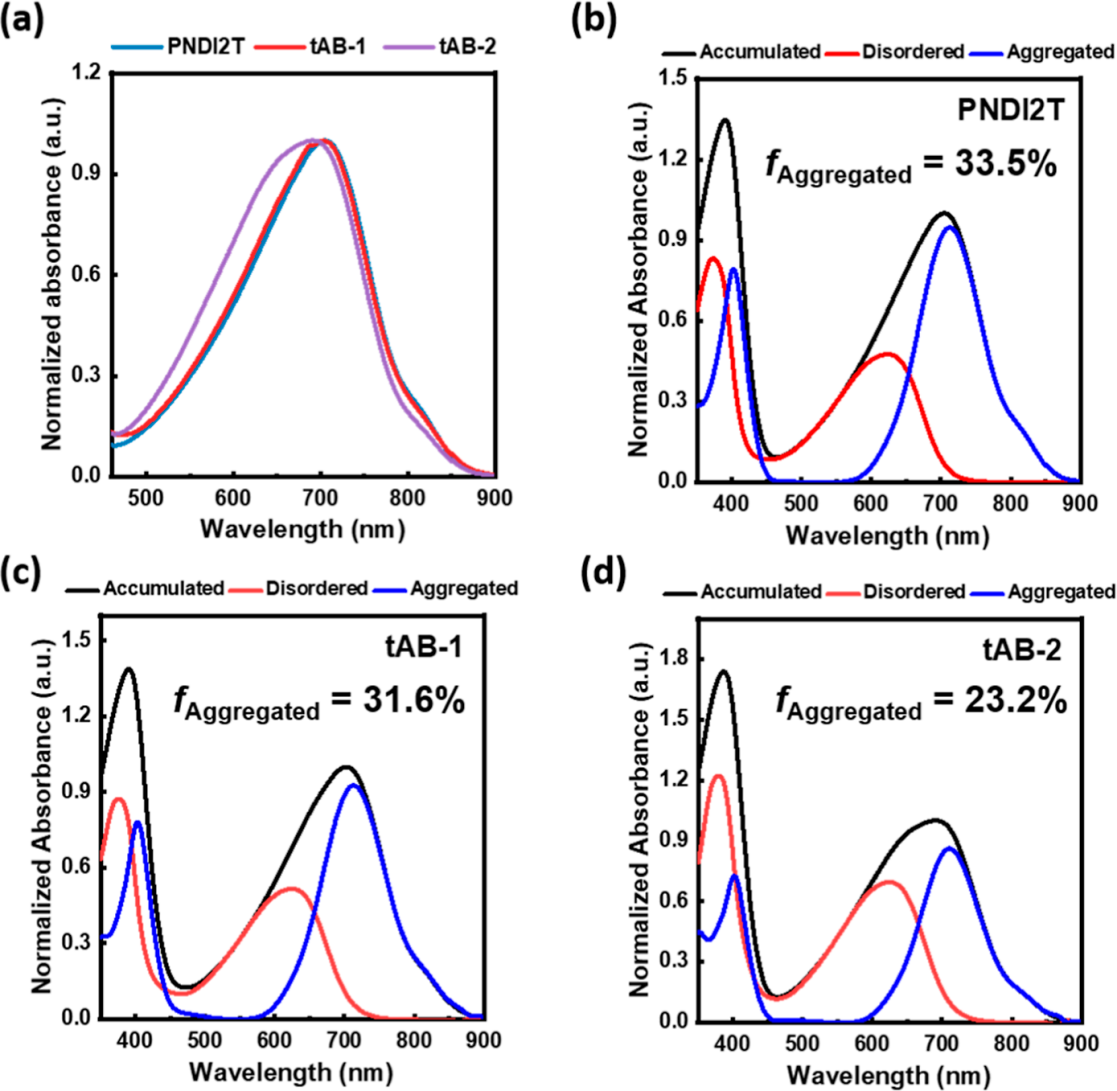
(a) Normalized UV–vis–NIR optical absorption spectra of the polymer solutions. Fraction of aggregation and disorder observed from the optical absorption spectra of (b) PNDI2T, (c) tAB-1, and (d) tAB-2 defined in toluene and 1-chloronaphthalene, respectively.

### Optical Characterizations of the Polymer/S-SWCNTs

The sorting performance is characterized using optical analyses, including the optical absorption and Raman spectroscopy. Figure S12 and Supporting Information describe the detailed preparation of the polymer/s-SWCNT composite. To analyze the sorting results, the polymer signal is subtracted from the UV–vis–NIR spectra. The sorting yield of s-SWCNTs was calculated using Beer’s law (*A* = ε*bc*), where the extinction coefficient ε is 11.7 cm^2^/mg.^40,41^ The sorting yield can be expressed as yield = (c_s‑SWCNTs_ *V*_sorting_)/(*W*_unsorted SWCNTs_). The purity index Φ is determined from the *S*_22_ peak intensity (*A*_S22_/(*A*_S22_ + baseline)).^42^ A Φ value greater than 0.41 indicates a purity level above 99.9%.^43^ Figure 2a–c summarize the sorting performance of these polymers. The sorting yield for tAB-2 is higher than that of other polymers because its reduced self-aggregation facilitates a better interaction with the nanotubes. Since all composite materials exhibit Φ values >0.41, the samples contain high-purity s-SWCNTs (>99.9%).

**Figure 2:**
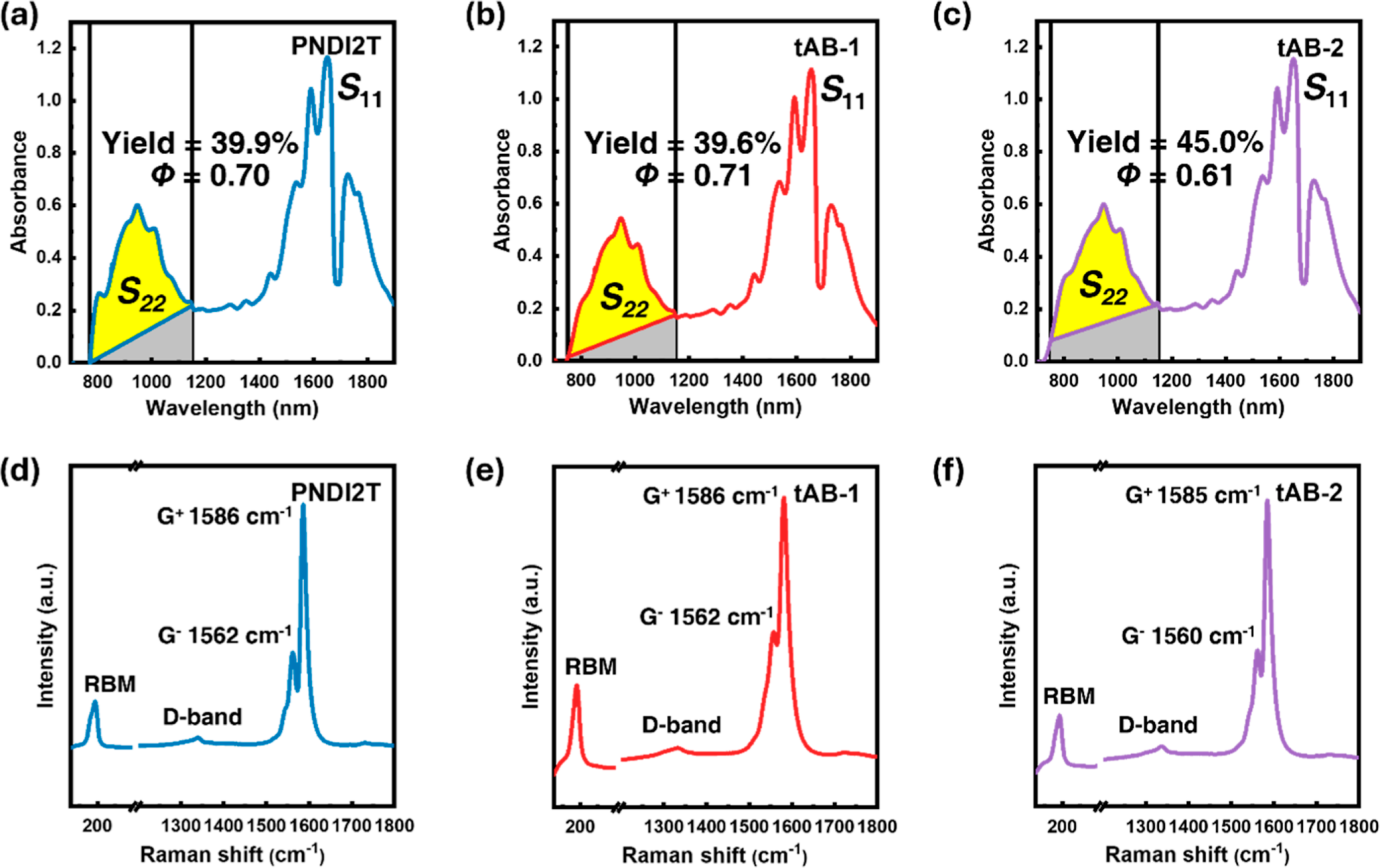
UV–vis–NIR optical absorption spectra of the polymer/s-SWCNT sorting solutions: (a) PNDI2T, (b) tAB-1, and (c) tAB-2. Raman spectra of the drop-cast polymer/s-SWCNT thin film using 532 nm laser: (d) PNDI2T, (e) tAB-1, and (f) tAB-2.

Figure 2d–f show the Raman spectra for each composite. The spectra display the G-band (1500–1600 cm^–1^), D-band (1300–1400 cm^–1^), and the radial breathing mode (RBM, ∼200 cm^–1^). Three critical parameters are obtained from these data: the G^+^/G^–^ ratio, the G/D ratio, and the calculated diameter *d*_RBM_. Note that the G^+^/G^–^ ratio was calculated using a Lorentzian function for deconvolutional fitting of the G^+^ and G^–^ bands.^44,45^ The G^+^/G^–^ ratio identifies the SWCNT type. s-SWCNTs show a high G^+^/G^–^ ratio because the G^+^ peak intensity is significantly larger than the G^–^ peak. In contrast, metallic nanotubes show a significantly higher G^–^, thereby lowering the G^+^/G^–^ ratio. The G^+^/G^–^ ratios for PNDI2T, tAB-1, and tAB-2 composites are 3.22, 3.09, and 3.32, respectively. These values confirm that all polymers studied have excellent selectivity for s-SWCNTs.

Next, the G/D ratio indicates the defect density within the composite.^42^ A higher G/D ratio indicates fewer defects. The D-band intensity is indicative of sidewall defects or carbon impurities.^46^ Because of their high purities, the G/D ratio directly reflects the quality of the SWCNT sidewalls. The G/D ratios for PNDI2T, tAB-1, and tAB-2 composites are 9.22, 8.58, and 8.21, respectively. The tribranched design yields a lower G/D ratio than PNDI2T. In addition, defects may originate from both the synthesis and sonication processes, in which high power can cause tube breakage.^47^ Among these, the tAB-2 composite possesses optimal defects that may benefit memory device performance. Finally, the s-SWCNT diameter was calculated from the RBM frequency ω_RBM_ using the formula: ω_RBM_ = *A*/*d*_RBM_ + *B*, where *A* = 248 nm cm^–1^ and *B* = 0 cm^–1^.^48^ Based on the sorting environment with polymer wrapping, the composite exists in a debundled form. This debundled state is later confirmed via the height profile of AFM images. The *d*_RBM_ values for PNDI2T, tAB-1, and tAB-2 composites are 1.282, 1.310, and 1.292 nm, respectively. The tribranched architecture successfully captures nanotubes with larger diameters. Typically, larger diameters correlate with fewer defects due to a lower strain. However, the observed G/D ratios and diameters show a complex relationship, as multiple factors influence the D-band intensity.^49–52^

### Chirality Characterizations of the Polymer/S-SWCNT Sorting

PLE analysis was applied to investigate the chirality distribution of the sorted s-SWCNTs. Figure 3a–c show the 2D PLE maps for PNDI2T, tAB-1, and tAB-2 composites, respectively. Using the Kataura plot^53^ and correcting for solvent effects,^54^ the (*n*, *m*) chiral indices to the s-SWCNTs are listed in the figures. Each chirality corresponds to a specific nanotube diameter. According to Jorio et al., the s-SWCNT diameter was fitted using the formula: $d=\left(a\sqrt{n^{2}+m^{2}+nm}\right)/\pi$ , where the lattice constant (*a*) is 0.246 nm.^55^ The PD SWCNTs exhibit a diameter range from 0.9 to 1.5 nm. This range is divided into three categories: small (0.9–1.1 nm), medium (1.1–1.3 nm), and large (1.3–1.5 nm) diameters. Figure S13 shows the ratio of each chirality, which is calculated from the fluorescence emission power *F*. The relationship follows the equation *F* = 2.303 ϕ_F_ *K*″ ε*bc P*_0_. In this calculation, we assume the fluorescence quantum yield ϕ_F_ is constant for all chiralities due to the difficulty in determining the individual ϕ_F_ of s-SWCNT with different chiralities. To find the extinction coefficient ε, an empirical formula derived by Sanchez et al. was applied to determine the absorption cross-section σ_S11_ = (0.92 + 0.81e^(0.85–*d*)/0.158^)*10^–17^ (cm^2^/atoms).^56,57^ Then, σ_S11_ was converted to ε_S11_ using the molar mass relation ε_S11_ = (σ_S11_*N_AD_)/*M*_C_*10^–3^ (cm^2^/mg), where *N*_AD_ is the Avogadro’s constant and *M*_C_ is the atomic mass of carbon.^58^ From these calculations, the average diameter from PLE analysis (*d*_PLE_) can be obtained. The *d*_PLE_ values for PNDI2T, tAB-1, and tAB-2 composites are 1.204, 1.197, and 1.195 nm, respectively. Figure 3d illustrates the diameter distribution for each composite. These results differ significantly from the *d*_RBM_ values derived from Raman spectroscopy (*d*_RBM_). This discrepancy arises because the PLE measurement range is primarily limited to small- and medium-diameter s-SWCNTs. Introducing soft segments into the polymer backbone results in a broader diameter distribution of the sorted s-SWCNTs. The soft segments increase backbone flexibility, allowing the polymer to wrap s-SWCNTs of diverse diameters. This flexibility likely enables the polymer to sort not only small diameters but also large diameters that fall outside the PLE detection limit, considering the deviation between *d*_PLE_ and *d*_RBM_. To better contextualize the sorting efficiency, dispersion yield, and selectivity achieved by the tribranched and molecular claw-like architecture, a comprehensive comparison summarizing the sorting performance of various reported n-type polymer systems in the literature is provided in Table 2. The optimized tAB 2 composite delivers exceptional semiconducting purity alongside a competitive yield, establishing a robust material foundation for the subsequent high-performance optoelectronic memory devices.

**Figure 3:**
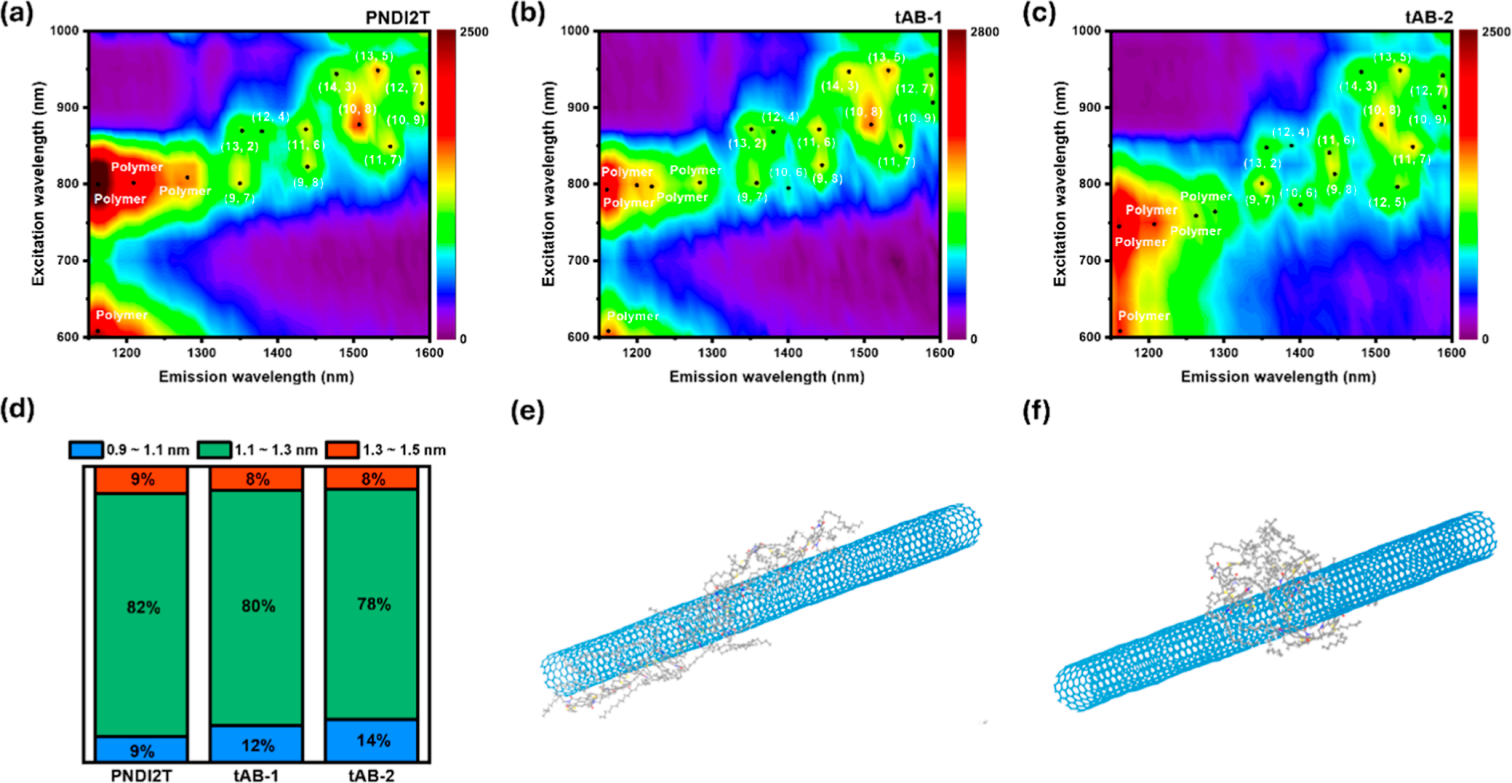
PLE spectra of the polymer/s-SWCNT sorting solutions: (a) PNDI2T, (b) tAB-1, and (c) tAB-2. (d) Diameter distribution of the s-SWCNTs calculated from the chirality distributions obtained from the PLE spectra. MD simulations of the polymer/s-SWCNT: (e) PNDI2T and (f) tAB-architectured polymer wrap around (10, 8) s-SWCNT. Note that (10, 8) is the major chirality derived from the conjugated polymer sorting in this study.

**Table 2:** Summary of s-SWCNT Sorting Performance Using Various Reported n-Type Conjugated Homopolymers and Block Copolymers and SWCNT Sources Including the PD, CoMoCAT, HiPco, and the CVD Synthesized

polymer/SWCNT type	sorting yield (%)	sorting purity (%)	diameter selectivity (nm)	refs
PNDI-2T/PD	18.3	>95	1.27	^50^
PhPDI/CoMoCAT	--	--	∼0.9	^32^
PS-*b*-PFO-*b*-PS/HiPco	0.05	--	0.82–1.09	^42^
PFO-*b*-PI/CVD synthesized	--	--	0.83–1.10	^43^
PCL-*b*-PF/CVD synthesized	20.0	∼90	1.145	^44^
tAB-2/PD	45.0	>99.9	1.09–1.30	this work

To investigate the conformational effect of the tribranched architecture, molecular dynamics (MD) simulations were applied. The detailed simulation procedure is described in the Supporting Information. Figures 3e,f and S14a–f display the simulation results for polymers sorting the (10, 8) s-SWCNT, which shows the highest abundance in the PLE spectra. The binding energy *E*_b_ of PNDI2T composite is −616 kcal/mol. However, the *E*_b_ of the tAB series composite is only −437 kcal/mol. The relatively lower *E*_b_ of tAB can be attributed to the lower content of the conjugated polymer component. The PNDI2T backbone binds more strongly than the tribranched conformation. This result may imply a lower Φ and a higher yield for tAB-2 due to weaker binding than that of the homopolymer. To further probe the diameter-dependent selectivity, MD simulations were extended to a smaller diameter (9, 7) of s-SWCNT, and the results are shown in Figures S14g–l. Interestingly, the *E*_b_ of the tAB series on the smaller tube increases significantly to −604 kcal/mol, representing a massive binding energy drop of 167 kcal/mol, whereas the linear PNDI2T exhibits only a modest shift to −695 kcal/mol. This sharp thermodynamic contrast underscores that the tribranched architecture is uniquely optimized to grasp smaller diameter nanotubes, in excellent agreement with the enrichment trends verified by PLE mapping. Morphologically, the stable structure in Figure S14k reveals that the conjugated blocks closely anchor onto the nanotube, while the soft PIB segments provide critical interfacial elasticity to adaptively conform to the highly curved surfaces of smaller s-SWCNTs, thereby enabling exceptional structural compliance and selection purity.

### Morphological Characterizations of the Polymer/S-SWCNT Thin Films

After investigating the polymer/s-SWCNT solutions, the thin-film morphology is subsequently characterized. Figure 4a–c display the thin-film morphology of the composite materials. The atomic force microscopy (AFM) images reveal distinct fiber structures. A topographic mapping analysis was applied to trace the fibers and to extract their lengths and widths. Considering the AFM tip convolution effect, the fiber width values in the lateral directions are limited to 10 nm.^59^ To accurately characterize the true dimensions of these hybrids and avoid the overestimation inherent to the lateral tip convolution effect, cross-sectional height profile analysis was employed. The individual fibers shown in Figure 4d–f exhibit a highly uniform vertical dimension, consistently ranging from 4 to 5 nm. This perfectly corresponds to the dimension of an individual s-SWCNT core encapsulated by a supramolecular polymer wrapping shell. This vertical metrology unambiguously demonstrates that the sorted carbon nanotubes are well separated and debundled within the polymer matrix. This is in excellent agreement with the sharp, well-resolved electronic transition peaks observed in the UV–vis–NIR absorption spectra in Figure 2a–c, which show the distinct peaks of the well-separated SWCNT.

**Figure 4:**
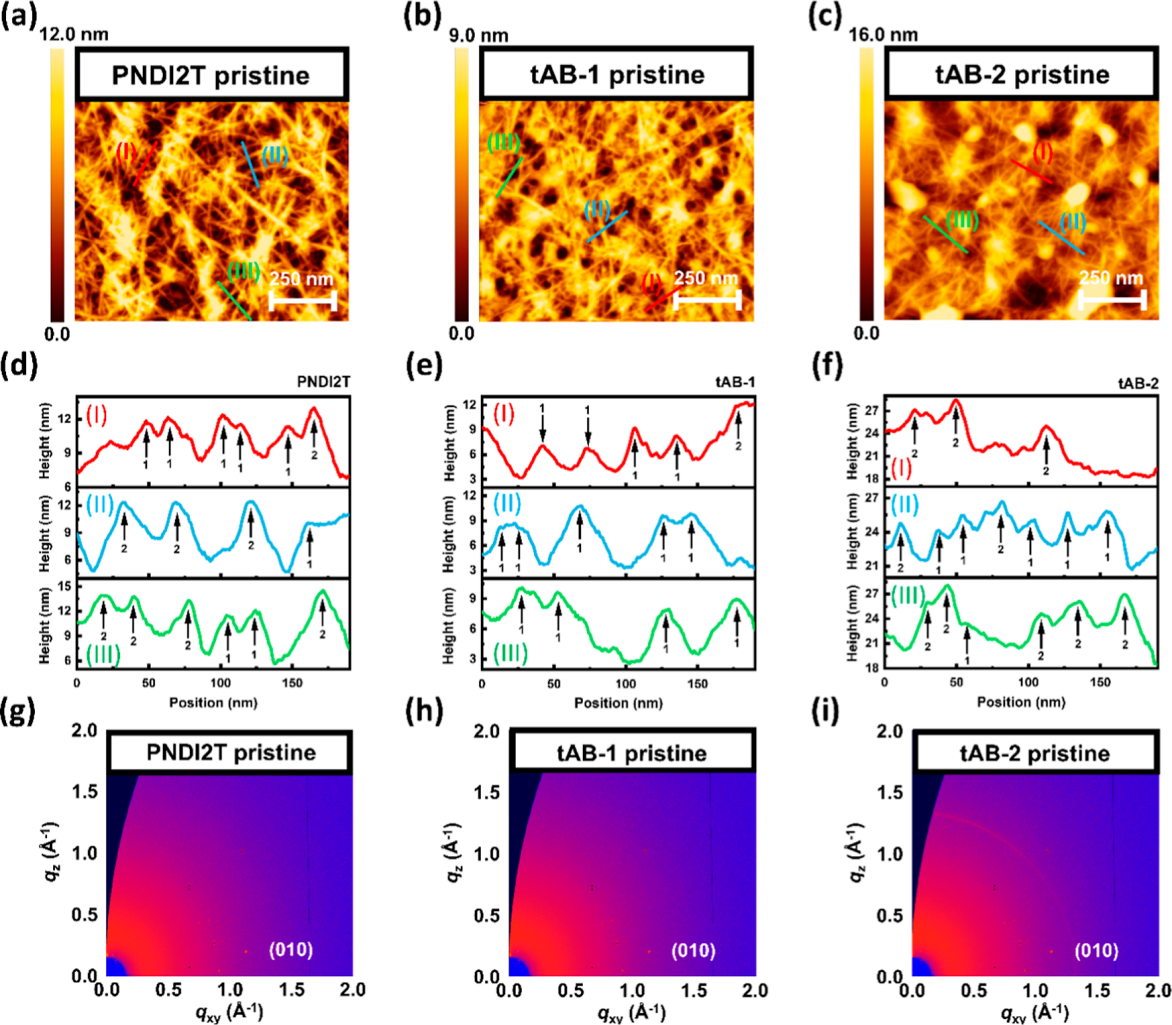
(a–f) AFM images with the height profile analysis and (g–i) 2D GIWAXS patterns of the polymer/s-SWCNT thin films: (a,d,g) PNDI2T/s-SWCNTs, (b,e,h) tAB-1/s-SWCNTs, and (c,f,i) tAB-2/s-SWCNT thin films. Note that the (I–III) labeled in (a–f) indicates the line-cutting height profiles, and the 1 and 2 labels in (d–f) indicate the single and stacked s-SWCNT tubes.

GIWAXS analysis can be applied to investigate the solid-state stacking and dispersion of the polymers in the composite films. Figures 4g–i and S15 show the 2D patterns and 1D line-cutting profiles used to evaluate the thin-film crystallinity of the composite materials. The films exhibit only a (010) reflection in the in-plane (IP) direction, corresponding to the π–π stacking of the polymer backbone along the nanotube axis. The *d*-spacings for PNDI2T, tAB-1, and tAB-2 films are 4.15, 4.14, and 4.50 Å, respectively. The paracrystalline disorder values for PNDI2T, tAB-1, and tAB-2 are 0.23, 0.23, and 0.26, respectively. The increased *d*-spacing and disorder in tAB-2 suggest an inferior structural order, likely induced by its highly hindered and soft-segment content. Concerning the perspective of device applications, this disorder and loose packing may create abundant trapping sites, forming an ideal nanomorphology for charge trapping and retention.

### Photomemory Characterizations of the Polymer/S-SWCNT Devices

After investigating the thin-film morphologies, the device characteristics were finally analyzed. Figure S16 details the device fabrication process. Figure 5a presents the schematic of the photomemory device. The hole-transporting s-SWCNTs and the electron-trapping polymers are integrated into a single active layer in an FET device. Figure S17 displays the transfer and output curves of the FET devices operating at a drain voltage *V*_D_ of −10 V. Both tAB-1 and tAB-2 composites exhibit a significant hysteresis in the transfer curves. This hysteresis confirms the presence of accessible charge trapping sites, which are essential for memory operation. Figure 5b–d present preliminary tests to evaluate the memory characteristics. An electrical writing (EW) operation with a *V*_G_ = 100 V pulse for 1 s, and an electrical erasing (EE) operation with a *V*_G_ = −100 V pulse for 1 s were applied to modulate the conductance of the memory device. The memory window (Δ*V*_TH_) and the memory ratio (*I*_ON_/*I*_OFF_) were extracted from the measured transfer curves. Figure S18a–c show the operations of EW (*V*_G_ = 100 V, 1 s) and optical erasing (OE, 254 nm UV light, 90 s). The erased OFF-state current after OE is obtained from the resulting transient current curves. The tAB-2 composite exhibits the largest Δ*V*_TH_ and the highest *I*_ON_/*I*_OFF_. These preliminary tests indicate that the tAB-2 composite possesses the highest performance for photomemory applications. Therefore, the subsequent discussion focuses primarily on the photomemory devices fabricated with the tAB-2 composite.

**Figure 5:**
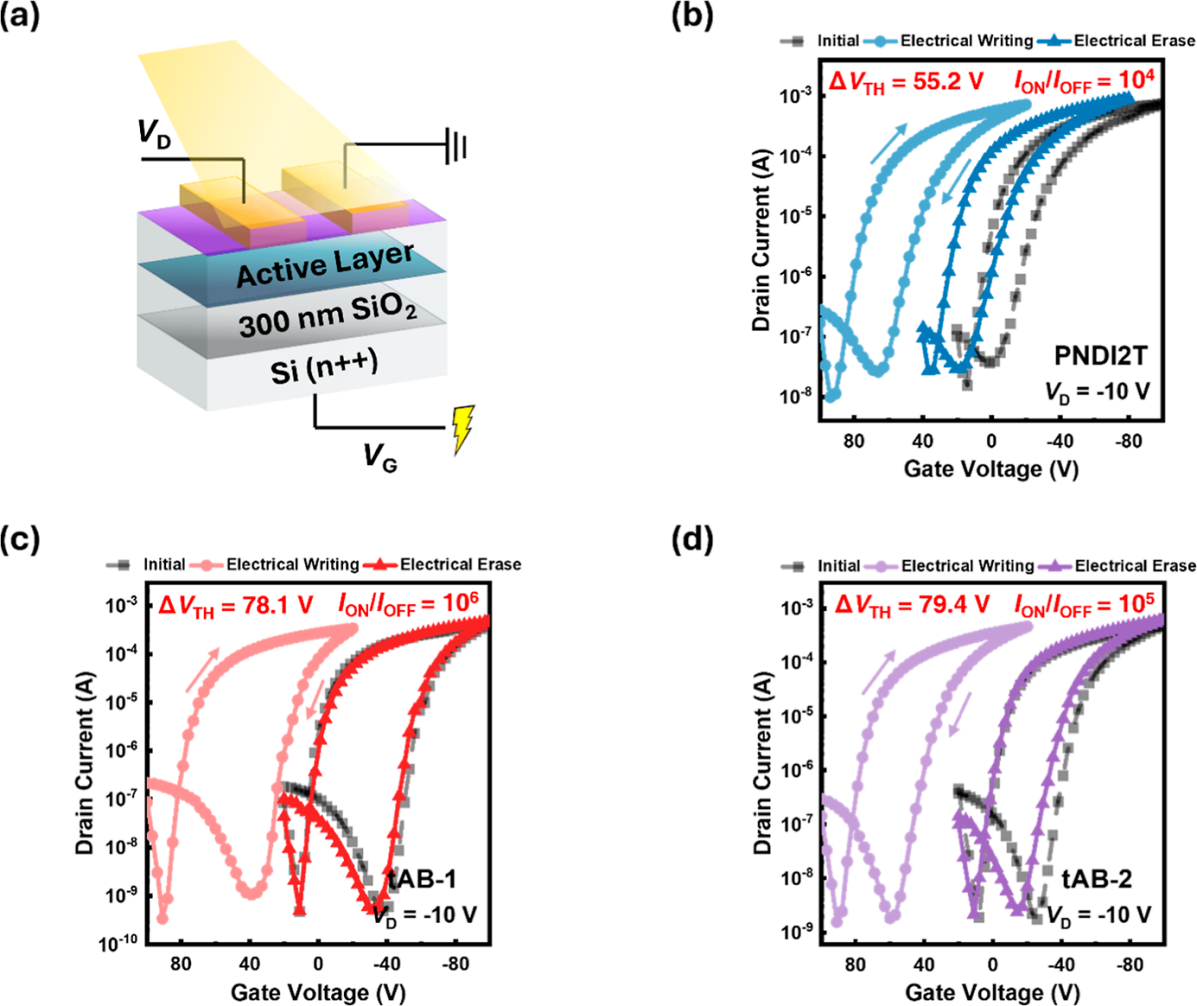
(a) Schematic of the photomemory device with a polymer/s-SWCNT channel as the active layer for carrier transport in the FET and counter charge trapping to the memory behavior. Photomemory characteristics evaluation under electrical writing and electrical erasing operations of polymer/s-SWCNT devices: (b) PNDI2T, (c) tAB-1, and (d) tAB-2. Note that the electrical writing and erasing were conducted by applying *V*_G_ = 100 and −100 V at *V*_D_ = 0 V for 1 s, respectively; the transfer curves were measured at *V*_D_ = −10 V. The sweeping rate is 16 V/s and the range of the transfer curve of the initial state is 20 to −100 V, the electrical writing state is 100 to −20 V, and the electrical erasing state is 20 to −100 V, except that the PNDI2T electrical erasing state is 40 to −80 V.

To gain a deeper understanding of the underlying physics of this photomemory behavior, the mechanism and explicit roles of the active components during programming are illustrated in Figure 6. In this design, the s-SWCNT network acts as the hole-transporting channel, whereas the tAB-2 copolymer functions as a dual-purpose light-absorbing and charge-trapping electret layer.^40^ The EW state is achieved by applying a positive gate bias that injects electrons into the polymer matrix. Because of the significant energy-level mismatch and potential barrier introduced by the insulated PIB domains, these injected carriers are effectively trapped and stored in localized trap states rather than passing through. The resulting stable accumulation of negative charges generates a persistent internal electric field that capacitively gates the s-SWCNT channel, keeping the device in a high-conductance ON state. To reset the memory, an EE pulse can be applied to detrap or neutralize the stored electrons. Alternatively, the device features a distinct OE mechanism in which light illumination generates excitons within the polymer matrix; these photogenerated holes readily overcome the local barrier to recombine with trapped electrons, thereby restoring the s-SWCNT channel to its original low-conductance OFF state and completing the nonvolatile memory cycle.

**Figure 6:**
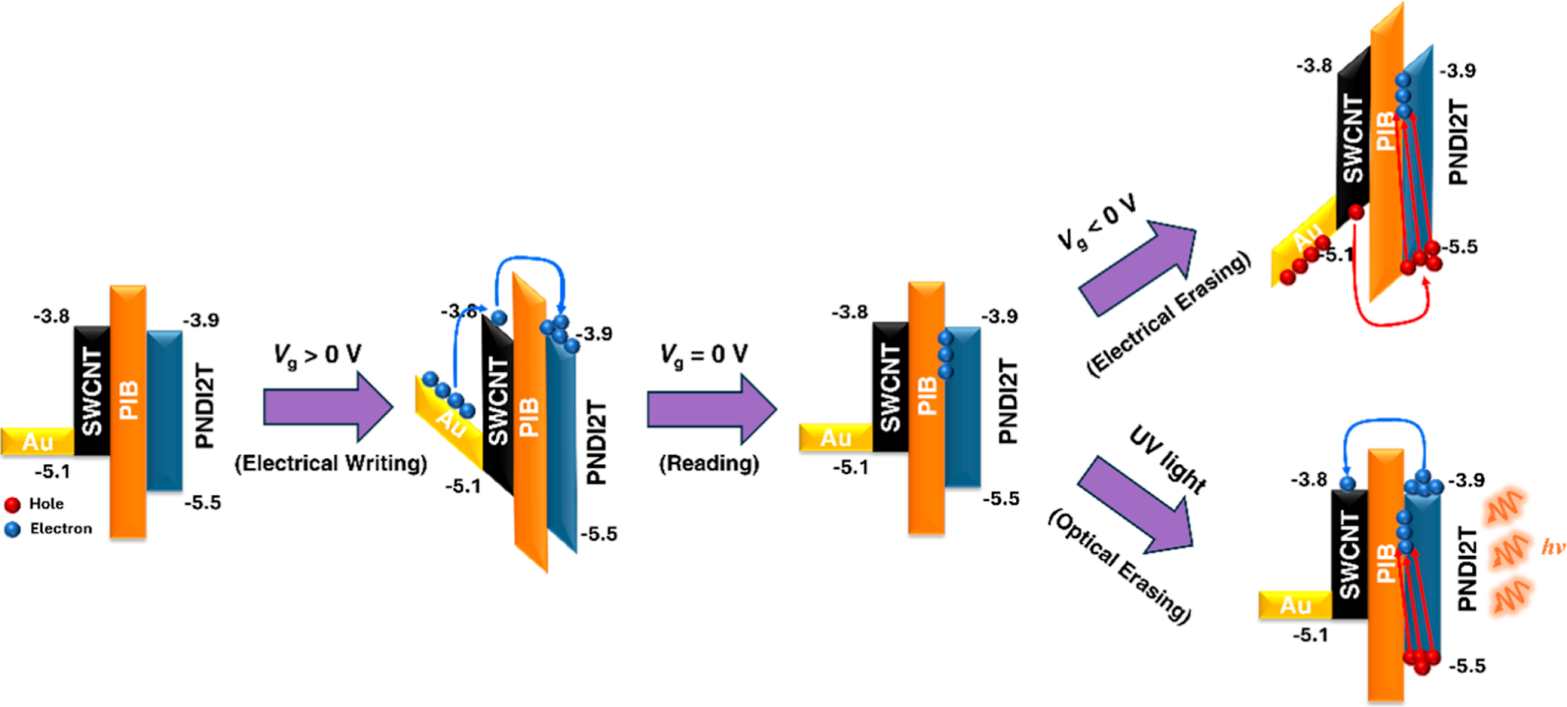
Working mechanism of the photomemory comprising the polymer/s-SWCNT channel with electrical writing to the ON-state and electrical or optical erasing to the OFF-state.

### Durability and Multilevel Characterizations of the Photomemory Devices

Photomemory devices require nonvolatile data retention, cycling endurance, and stable multilevel operation.^60–62^ First, a cycling test was conducted with the EW and OE operations at a drain voltage *V*_D_ of −10 V. Figure 7a shows that the device maintains its performance over 12 stepwise switching cycles, indicating excellent stability. Notably, the memory device can be operated effectively at a low *V*_D_ of −10 mV and maintains a distinct memory ratio (>10^3^, Figure S18d). Figure 7b presents the OE process results under various light wavelengths for 90 s at *V*_D_ = −10 mV. The 254 nm UV light exhibits the highest erasing efficiency, recovering the device to its lowest OFF-state current. Figure 7c illustrates the retention test for the tAB-2 composite device. The ON-state transient curve is measured after the EW operation, and the OFF-state curve is measured after the OE operation. The single-layer design makes the device susceptible to memory-state loss, particularly in the OFF state. Ambient oxygen acts as a p-dopant for s-SWCNTs, increasing the OFF-state current over time.^63^ Despite this oxygen doping effect, the device maintains a distinguishable memory ratio after 20,000 s. Concerning the memory endurance, Figure 7d shows the write–read–erase–read (WRER) test. The consecutive EW and OE operations were applied with a 40 s reading time after each step at *V*_D_ = −10 mV. The device undergoes 10 consecutive WRER cycles. The consistent current levels confirm the device’s high operational stability. The results indicate that the photomemory comprising the polymer/s-SWCNT channel is strong for short-term and dynamic data storage but weak for long-term storage. The constraint on long-term retention can be closely linked to the substantial amount of residual polymers in the channel layer. Excess insulating residues inevitably generate disordered defect states at the interface between the composite film and the dielectric layer, thereby simultaneously reducing charge transport efficiency. These adverse interfacial defects may increase the electronic tunneling barriers and form unstable, shallow trapping paths that facilitate gradual charge leakage over time, fundamentally accounting for the volatile nature and the lack of ideal long-term memory retention in the current device architecture.

**Figure 7:**
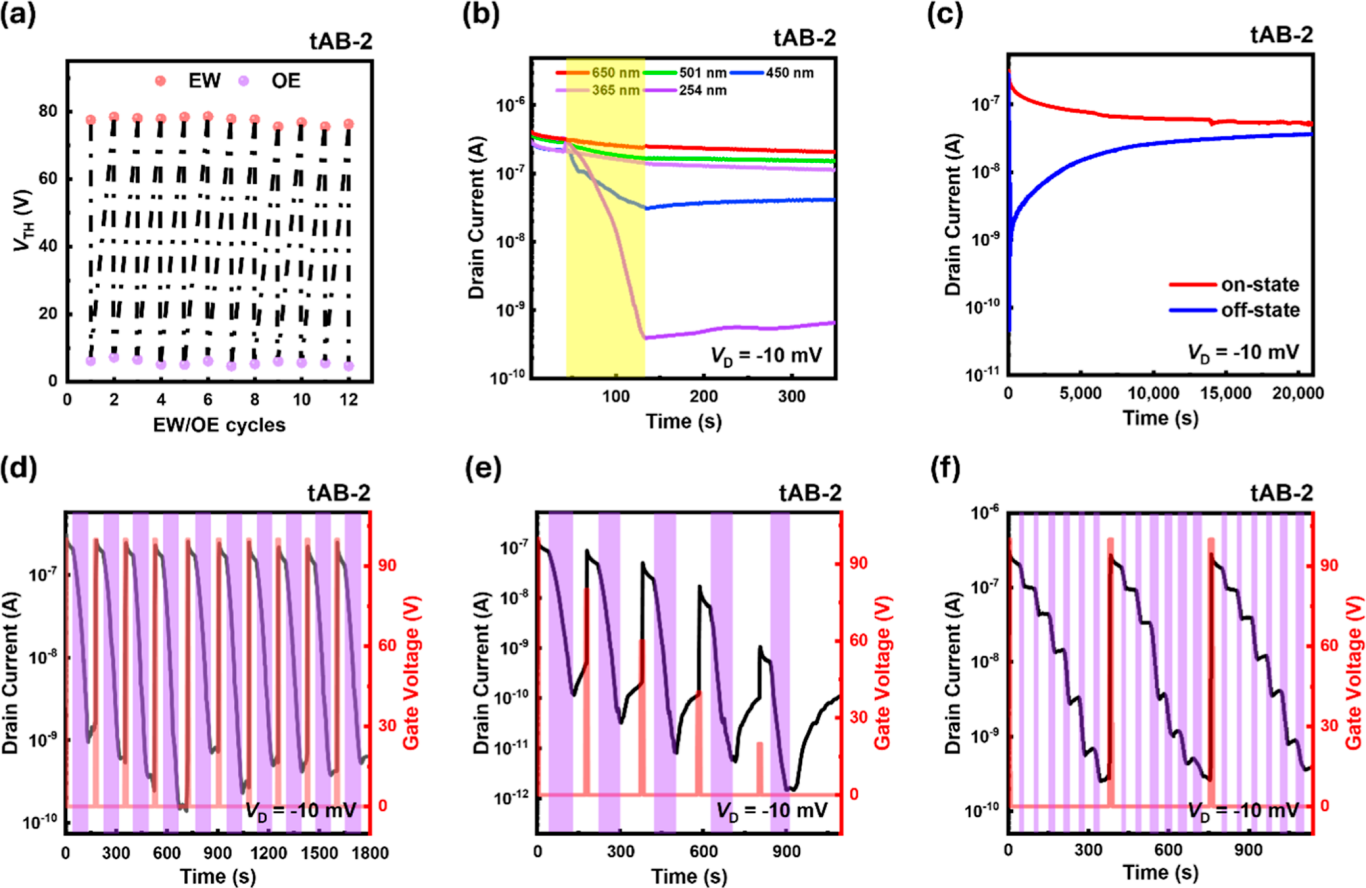
(a) Cycle operation of the tAB-2/s-SWCNT photomemory device with stepwise electrical writing and optical erasing. (b) Optical erasing performance by different light wavelengths for the tAB-2/s-SWCNT photomemory device. (c) Retention test, (d) WRER test, (e) multilevel electrical writing test, and (f) multilevel optical erasing test for the tAB-2/s-SWCNT photomemory device. Note that the transient current characteristics were measured at *V*_D_ = −10 mV, the electrical writing was conducted by applying *V*_G_ = 100 for 1 s, and the optical erasing was conducted by applying 254 nm light for different durations as indicated by the purple-shaded area.

For efficient dynamic data storage, multilevel memory operations are crucial. Accordingly, Figure 7e,f displays the multistep operation tests. Figure 7e shows the multistep EW test. This test combines programmed EW pulses with a constant 90 s OE operation using 254 nm UV light. The programmed EW operation applies varying *V*_G_ values of 100, 80, 60, 40, and 20 V in consecutive cycles to achieve distinct multilevel on-states. Figure 7f presents the multistep OE test. This test uses a constant EW pulse (*V*_G_ = 100 V), followed by different OE operations. The programmed OE operation divides the 90 s erasing process into six 15 s segments to demonstrate discrete multilevel OFF-states triggered by the accumulated light exposure. A reading process occurs after each 15 s OE segment. The results from both multistep tests demonstrate excellent current discrimination and high stability under variable operating conditions. These tests confirm that the tAB-2 composite photomemory device possesses an excellent overall performance. The dynamic operation and multilevel capability provide a significant advantage for practical multibit storage applications.

## Conclusion

Inspiring by the molecular topology for s-SWCNT sorting, n-type semiconducting tribranched multiblock copolymers are developed by incorporating PIB soft segments into a rigid NDI2T backbone. Indeed, two polymers, tAB-1 and tAB-2, were investigated and compared against a linear control, PNDI2T, to evaluate their s-SWCNT sorting capabilities and photomemory performance. Although the tribranched multiblock copolymers have a hyperbranched rather than an exact claw/star architecture, this molecular claw-like topology increases the contact area with the nanotube surface. The high ratio of soft segments in tAB-2 significantly disrupted the backbone planarity and yielded the lowest polymer self-aggregation fraction of 23.2%, enhancing solubility and facilitating superior interactions with the nanotubes. Consequently, tAB-2 achieves an outstanding sorting purity exceeding 99.9% (Φ > 0.41). The topology effect enhances interactions and facilitates the capture of small-diameter s-SWCNTs. These small-diameter nanotubes intrinsically possess higher defect densities. Raman spectroscopy confirms this trend. The tAB-2 composite exhibits a lower G/D ratio of 8.21 than the linear control. This lower ratio signifies a higher defect density in the sorted nanotubes. Structural analysis reveals that the tAB-2 composite forms debundled fibers with an increased paracrystalline disorder. This loose molecular packing generates essential charge trapping sites. Thus, the tAB-2 polymer exhibits an excellent trade-off between sorting purity and defect density. Rather than degrading performance, this intentionally induced loose packing creates abundant, accessible charge-trapping sites at the polymer/s-SWCNT heterojunction. Consequently, the tAB-2 photomemory exhibits exceptional characteristics. It achieves a wide Δ*V*_TH_ of 80 V and a high memory ratio of 10^5^. The device demonstrates excellent dynamic operational stability. It features distinct multilevel states modulated by programmed EW and OE operations. All states are readable at an ultralow *V*_D_ of −10 mV to minimize power consumption. The results indicate that the polymer/s-SWCNT photomemory excels in short-term and dynamic data storage. While long-term retention is limited, the device demonstrates robust performance in dynamic operations. Ultimately, this study establishes a vital molecular design guideline. Utilizing topological engineering, such as the hyperbranched, star-shaped, and molecular claw-like, are versatile in balancing polymer flexibility, sorting efficiency, and interfacial defect engineering. This strategy provides a clear path for advancing next-generation organic optoelectronic memory.

## Experimental Section

### Materials

1,3,5-Tris­(2-chloropropan-2-yl)­benzene^64,65^ and PNDI2T^21^ (number-average molar mass (*M*_n_) = 66,200 g/mol, molar-mass dispersity (*D̵*_M_) = 1.69) were synthesized according to previously reported procedures. Plasma-discharged single-walled carbon nanotubes (PD-SWCNTs, >90% carbon basis), poly­(methyl methacrylate) (PMMA, *M*_w_ ∼ 350,000 g/mol), polystyrene-*b*-polybutadiene-*b*-polystyrene (SBS, 30 wt % styrene), pentaerythritol tetrakis­(3-mercapto-propionate) (PETMP), and phenylbis­(2,4,6-trimethylbenzoyl)­phosphine oxide (BAPO) were purchased from Sigma-Aldrich. Poly­(styrene sulfonic acid) (PSS, *M*_w_ ∼ 45,000–70,000 g/mol) was purchased from Macklin. All chemicals and solvents were used as received without further purification.

### Characterizations

^1^H nuclear magnetic resonance (NMR) was documented using a JEOL JNM-ECX400 spectrometer at nuclear resonant frequencies of 400 or 600 MHz in chloroform-*d* (CDCl_3_) at 25 °C (for precursory PIB homopolymers) or in 1,1,2,2-tetrachloroethane-*d*_2_ (C_2_D_2_Cl_4_) at 100 °C (for NDI-2T based polymers). The *M*_n_, weight-average molar mass (*M*_w_), and molar-mass dispersity (*D̵*_M_) values of precursory PIB homopolymers were measured by size exclusion chromatography (SEC) using a JASCO GULLIVER HPLC system equipped with a pump (JASCO PU-4580), a column oven (JASCO CO-2065 Plus), and a RI detector (RI-1530). The column set was as follows: a guard column (Shodex K-G 4A) and two consecutive columns (Shodex K-804L, Shodex K-805L) eluted with tetrahydrofuran at 40 °C at a flow rate of 1.0 mL/min. The *M*_n_, *M*_w_, and *D̵*_M_ values of NDI-2T-based polymers were measured by SEC using a JASCO GULLIVER HPLC system equipped with a pump (JASCO PU-4580), a column oven (JASCO CO-1565), and a UV detector (UV, λ = 254 nm, JASCO UV-4575). The column set was as follows: a guard column (Shodex K-G 4A) and two consecutive columns (Shodex K-804L, Shodex K-805L) eluted with chloroform at 40 °C at a flow rate of 1.0 mL/min. Polystyrene standards were employed to prepare calibrations for all the SEC experiments. Thermal gravimetric analysis (TGA) was performed using TG/DTA6200 equipped with an EXSTAR 6000 (Hitachi High-Tech) with a heating rate of 10 °C/min under a nitrogen flow. Differential scanning calorimetry (DSC) analysis was conducted using DSC6200 equipped with an EXSTAR 6000 (Hitachi High-Tech) with a ramping rate of 10 °C/min under a nitrogen flow. Optical analysis of the polymer solutions were analyzed using UV–vis–NIR absorption spectroscopy on a JASCO V-770 spectrometer. Raman spectra of the polymer/s-SWCNT films were measured using a UniDRON spectrometer (CL Technology Co., Ltd.) with 530 nm excitation. The surface morphology of the polymer/s-SWCNT films was examined using an AFM100 plus (Hitachi) in the tapping mode at room temperature. The electrical performance of the fabricated FETs was measured using a Keithley 4200-SCS semiconductor parameter analyzer under ambient atmosphere.

### Solution Preparation

The SBS solution was prepared by dissolving SBS (10 mg/mL), BAPO (0.4 mg/mL), and PETMP (0.4 mg/mL) in toluene. The mixture was stirred overnight in the dark to ensure complete dissolution. The PMMA solution was prepared by dissolving PMMA in toluene at a concentration of 40 mg/mL. The PSS solution was prepared by dissolving PSS in deionized (DI) water at a concentration of 40 mg/mL.

### s-SWCNT Sorting Process

Five milligrams of the polymer were dissolved in 20 mL of toluene, followed by the addition of 10 mg of unsorted plasma discharged (PD) SWCNTs. The mixture was sonicated in an IPA ice bath at 70% power for 30 min. The work time of the tip sonicator is 5 s, followed by a 5 s rest. The solution was then centrifuged at 16,000 rpm (relative centrifugal force, RCF = 20,000g) for 60 min to collect the supernatant.

### Fabrication of Photomemory Devices

The device fabrication begins with the preparation of a sacrificial layer. A 1 wt % PSS-H solution was spin-coated onto a 300 nm SiO_2_ wafer and heated at 100 °C for 10 min. Next, the polymer/s-SWCNT solution was dropped onto the wafer. The film growth process proceeds for 2 h in a toluene vapor-rich environment. Afterward, the wafer was dipped in toluene to remove the excess polymer. Next, a 5 wt % PMMA solution was spin-coated onto the s-SWCNT film to serve as a support layer. The entire thin film is immersed in a water bath. The water dissolves the PSS-H layer, causing the PMMA/s-SWCNT film to detach from the substrate and float while still attached to the tape. Separately, a target substrate was prepared by spin-coating an SBS solution onto a fresh 300 nm SiO_2_ wafer. The SBS layer is cured under UV light. The floating s-SWCNT film was printed on the SBS-coated wafer. The wafer is heated at 60 °C overnight to ensure strong adhesion between the film and the substrate. Finally, the PMMA layer was removed by using acetone. The device fabrication is completed by thermally depositing the source and drain electrodes (30 nm thick gold) with a channel width and length of 1000 and 50 μm, respectively.

### MD Simulation

The polymer is prebuilt in Avogadro and then optimized using the MMFF94s force field. The carbon nanotube is built in the Material Studio, and the polymer is added to the file. The whole structure is optimized using the COMPASSII force field with Ewald electrostatics and atom-based van der Waals interactions. Then, the optimized file calculates the energy using the *NVT* ensemble at 300 K and 1000 ps, with a 1 fs time step and the COMPASSII force field, employing the Ewald electrostatics and atom-based van der Waals methods in ultrafine calculations. The nonbond energy *E*_nb_ includes the electrostatic and van der Waals. The binding energy *E*_b_ is calculated through the formula: ${E_{b}=E}_{nb,polymer/s‐SWCNTs}-E_{nb,polymer}-E_{nb,s‐SWCNTs}$ .

### Synthesis of tPIB-Br

All manipulations were performed in a glovebox unless otherwise noted. An initiator solution of 1,3,5-tris­(2-chloropropan-2-yl)­benzene (0.11 g, 0.35 mmol) dissolved in hexanes (1.67 g, 2.5 mL) was introduced into a 300 mL flask equipped with an evacuation tube fitted with a high-vacuum valve. Subsequently, 2,6-di-*tert*-butylpyridine (DTBP, 0.14 g, 0.70 mmol) and a solvent mixture of hexane (39 mL) and dichloromethane (18.9 mL) were added, and the flask was tightly sealed. In parallel, titanium tetrachloride (TiCl_4_, 0.48 g, 2.52 mmol) was placed in a 60 mL syringe vial, followed by the addition of hexane (5 mL) and dichloromethane (2.14 mL) (7:3, v/v), and the vial was sealed. In another 30 mL syringe vial, freshly distilled thiophene (16.6 mL, 210 mmol) was combined with hexanes (12.5 mL) and dichloromethane (5.4 mL) (7:3, v/v) and sealed. The reaction vessel and syringe vials were removed from the glovebox, and the reaction flask was cooled to −78 °C using a dry ice/acetone bath. Isobutene gas was first liquefied using a dry ice/acetone bath (−78 °C) and measured with a liquid nitrogen-cooled glass syringe. Liquefied isobutene (3.30 mL, 34.6 mmol) and then the TiCl_4_ solution from the syringe vial were added to the reaction flask to start the polymerization while maintaining the low temperature. The mixture was stirred at −78 °C for 1.5 h under a nitrogen atmosphere, after which a 2 mL aliquot was withdrawn. The thiophene solution was then rapidly injected into the reaction flask via a syringe, and the reaction was further stirred at −78 °C for 4.5 h. The reaction was quenched by the addition of methanol precooled to −78 °C, and the mixture was poured into a beaker containing methanol/aqueous ammonia (9:1, v/v, 100 mL). The resulting mixture was washed twice with water/isopropanol/NaCl (77.5:15:7.5, v/v/w) and twice with water. The crude product was purified by repeated dissolution–precipitation using hexane and methanol to afford three-armed star-shaped polyisobutene (PIB) bearing thien-2-yl end groups (tPIB-T) as a viscous liquid (2.34 g, 79%). ^1^H NMR (400 MHz, CDCl_3_, 25 °C, Figure S3): δ (ppm) 7.13 (s), 7.11–7.10 (m), 6.88 (dd), 6.79 (d), 1.41–1.39 (m), 1.11–1.03 (m).

Next, tPIB-T (0.5 g, 0.059 mmol) was dissolved in THF (7.4 mL) in a 100 mL two-neck flask and cooled to 0 °C in an ice bath. *N*-Bromosuccinimide (NBS, 0.32 g, 1.77 mmol) was then added, and the reaction mixture was stirred overnight. The solution was concentrated using a rotary evaporator, followed by the addition of hexane and stirring for 1.5 h. The precipitated insoluble fraction was removed by filtration, and the filtrate was concentrated again. The product was purified by dissolution–precipitation using hexane and methanol. Dimerized high molecular-weight species were removed by HPLC fractionation of the resulting polymer (Figure S1). The product was finally dried under high vacuum. The yield before HPLC purification was 0.39 g (76%), and after HPLC purification was 0.20 g (55%). *M*_n_ (SEC) = 8,600, *D̵*_M_ = 1.09. ^1^H NMR (400 MHz, CDCl_3_, 25 °C, Figure S2): δ (ppm) 7.13 (s), 6.82 (d), 6.54 (d), 1.46–1.39 (m), 1.16–1.05 (m).

### Synthesis of tAB-1

In a 50 mL two-neck flask, 4,9-dibromo-2,7-bis­(2-decyltetradecyl)­benzo­[lmn]­[3,8]­phenanthroline-1,3,6,8­(2*H*,7*H*)-tetraone (Br-NDI-Br, 0.10 g, 0.091 mmol) and 5,5′-bis­(trimethylstannyl)-2,2′-bithiophene (2T, 0.047 g, 0.095 mmol) were weighed and placed under a nitrogen atmosphere. After purging with nitrogen for 10 min, toluene (10 mL) was added. Following 30 min of nitrogen bubbling, Pd_2_(dba)_3_ (0.008 g, 0.009 mmol) and P­(*o*-tolyl)_3_ (0.028 g, 0.09 mmol) were introduced, and an additional portion of toluene (5.0 mL) was added. The reaction mixture was stirred under a reflux condition for 1 h (bath temp. = 120 °C). After monitoring the *M*_n_ of the resulting polymer (*M*_n_ (SEC) = 20,000, *D̵*_M_ = 2.08, Figure S4), by aliquot sampling, a solution of tPIB-Br (*M*_n_ (SEC) = 8600; *D̵*_M_ = 1.09, 0.029 g, 0.0034 mmol) in toluene (5.0 mL) was added to the reaction mixture. After 24 h, 2-(tributylstannyl)­thiophene (7 μL) was added, and the mixture was stirred for 2 h. Subsequently, 2-bromothiophene (4 μL) was added, and the reaction was continued for an additional 2 h. Sodium dimethyldithiocarbamate dihydrate (SDTC, Pd scavenger) was then added in an appropriate amount, and the reaction mixture was poured into methanol to precipitate the polymer. The obtained polymer was purified and recovered by Soxhlet extraction (methanol, acetone, hexane, and chloroform). Finally, a dark blue polymer, tAB-1, was obtained after freeze-drying from its benzene solution. Yield: 93 mg (72%). *M*_n_ (SEC) = 35,700, *D̵*_M_ = 3.51 (Figure S4). PNDI2T/PIB = 89:11 (w/w). ^1^H NMR (600 MHz, C_2_D_2_Cl_4_, 100 °C, Figure S6): δ (ppm) 8.86 (s), 7.39 (s), 4.16 (s), 2.04 (s), 1.20–1.70 (m), 1.17 (s), 0.89 (s).

### Synthesis of tAB-2

In a 50 mL two-neck flask, Br-NDI-Br (0.10 g, 0.091 mmol) and 2T (0.049 g, 0.100 mmol) were weighed and placed under a nitrogen atmosphere. After purging with nitrogen for 10 min, toluene (10 mL) was added. Following 30 min of nitrogen bubbling, Pd_2_(dba)_3_ (0.008 g, 0.009 mmol) and P­(*o*-tolyl)_3_ (0.028 g, 0.09 mmol) were introduced, and an additional portion of toluene (5.0 mL) was added. The reaction mixture was stirred under a reflux condition for 1 h (bath temp. = 120 °C). After monitoring the *M*_n_ of the resulting polymer (*M*_n_ (SEC) = 11,000, *D̵*_M_ = 1.73, Figure S5), by aliquot sampling, a solution of tPIB-Br (*M*_n_ (SEC) = 8600; *D̵*_M_ = 1.09, 0.068 g, 0.0079 mmol) in toluene (5.0 mL) was added to the reaction mixture. After 24 h, 2-(tributylstannyl)­thiophene (7 μL) was added, and the mixture was stirred for 2 h. Subsequently, 2-bromothiophene (4 μL) was added, and the reaction was continued for an additional 2 h. SDTC was then added, and the reaction mixture was poured into methanol to precipitate the polymer. The obtained polymer was purified and recovered by Soxhlet extraction (methanol, acetone, hexane, and chloroform). Finally, a dark blue polymer, tAB-2, was obtained after freeze-drying from its benzene solution. Yield: 93 mg (72%). *M*_n_ (SEC) = 36,400, *D̵*_M_ = 2.98 (Figure S6). PNDI2T/PIB = 66:34 (w/w). ^1^H NMR (600 MHz, C_2_D_2_Cl_4_, 100 °C, Figure S7): δ (ppm) 8.87 (s), 7.38 (s), 4.16 (s), 2.07 (s), 1.20–1.70 (m), 1.17 (s), 0.90 (s).

## Supplementary Material



## Notes

Synthetic routes of the conjugated polymers and the chemical structure characterizations; optical and electrochemical characterization of the polymer thin films; GIWAXS analysis of the thin films in the in-plane direction; topographic fitting results and parameters of the AFM morphologies; output curves of the phototransistor memory; and device fabrication process of the carbon nanotube transistors (PDF)
