# Supplementary material for: Tribranched Multiblock Copolymers Mimicking the Molecular Claw Design in N‑Type Conjugated Polymers for High-Yield Semiconducting Carbon Nanotube Sorting and Phototransistor Memory Applications
Source: ACS Appl Mater Interfaces. 2026 Jun 12;18(25):35776–89. doi: 10.1021/acsami.6c08012 (PMC13339005; doi:10.1021/acsami.6c08012)
Supplement: Supplementary file 1 [file am6c08012_si_001.pdf]

Supporting Information

Tribranched Multiblock Copolymers Mimicking the Molecular Claw Design in N-type Conjugated Polymers for High-Yield Semiconducting Carbon Nanotubes Sorting and Phototransistor Memory Applications

Yu-Che Kan,[a] *Shuto Yamamoto,*[b] *Yu-Chun Huang,*[a] *Yi-Hsuan Tung,*[a] *Guo-Hao Jiang,*[a]

Ming-Han Chen,[a] *Chien-Chung Shih,*[c] *He Sun,*[b] *Tomoya Higashihara,*[b*] *Yan-Cheng Lin* [a,d,e*]

[a] Department of Chemical Engineering, National Cheng Kung University, Tainan 70101, Taiwan

[b] Department of Organic Materials Science, Graduate School of Organic Materials Science, Yamagata University, 4-3-16 Jonan, Yonezawa, Yamagata 992-8510, Japan

[c] Department of Chemical Engineering and Materials Engineering, National Yunlin University of Science and Technology, Yunlin 64002, Taiwan

[d] Advanced Research Center for Green Materials Science and Technology, National Taiwan University, Taipei 10617, Taiwan

[e] Program on Smart and Sustainable Manufacturing, Academy of Innovative Semiconductor and Sustainable Manufacturing, National Cheng Kung University, Tainan 70101, Taiwan

[*]Corresponding author. E-mail: thigashihara@yz.yamagata-u.ac.jp (T. H.), ycl@gs.ncku.edu.tw (Y.-C. L.)

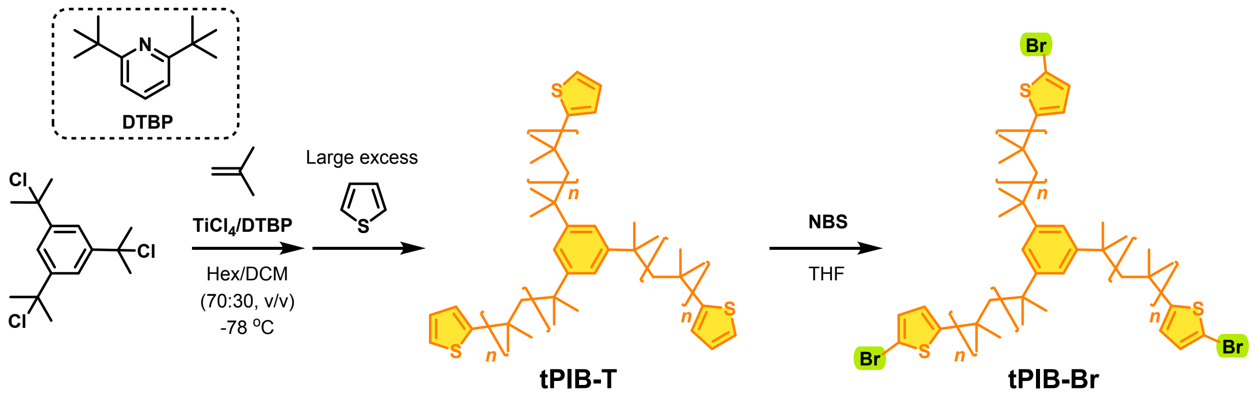

Scheme S1. Synthesis of tPIB-Br.

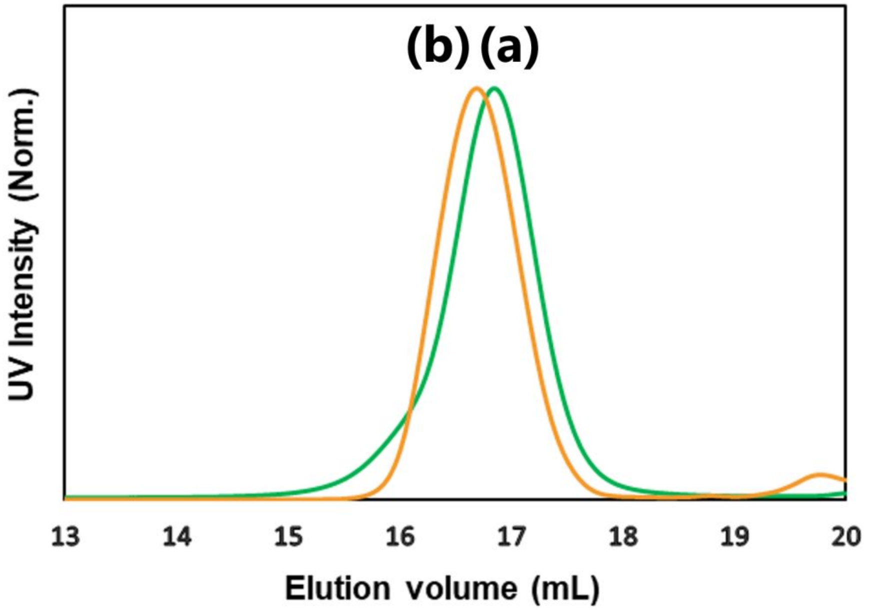

Figure S1. SEC UV traces of tPIB-Br: (a) before HPLC fractionation and (b) after HPLC fractionation.

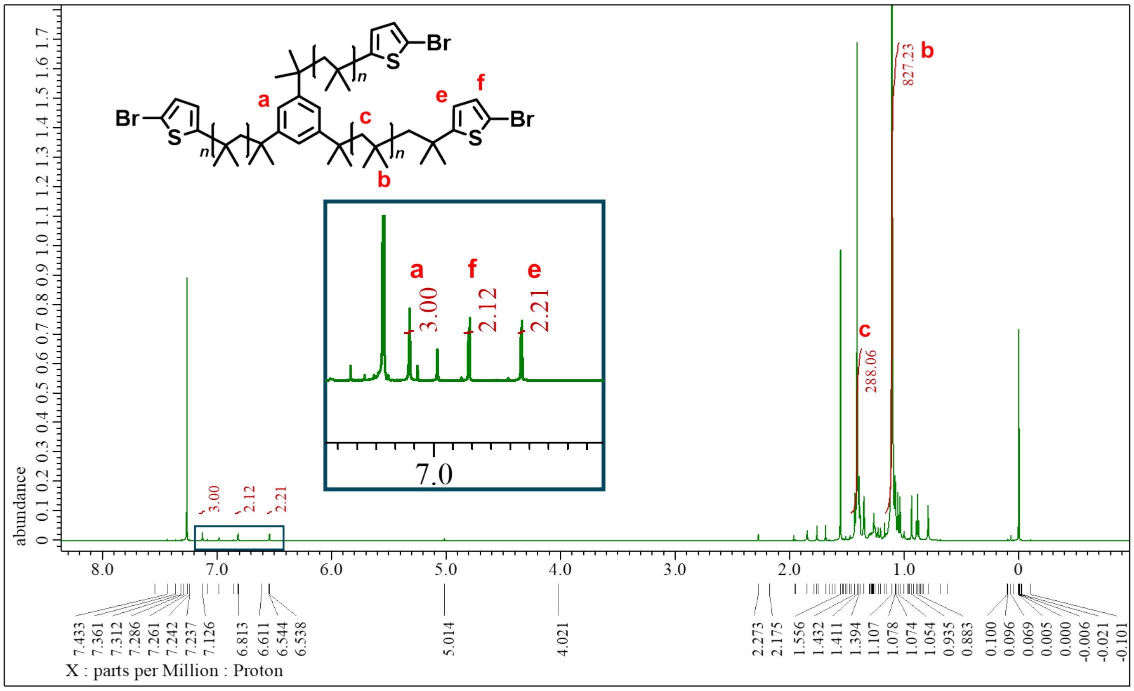

Figure S2. ^{1}H NMR spectrum of tPIB-Br in $CDCl_3$.

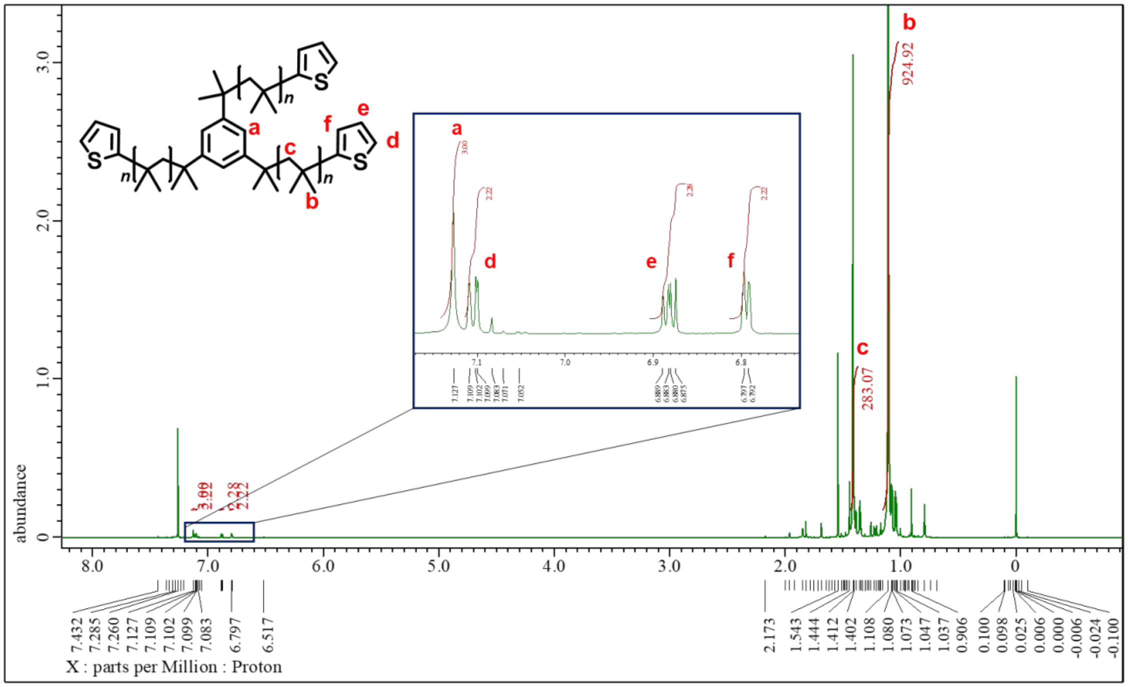

Figure S3. ^{1}H NMR spectrum of tPIB-T in $CDCl_3$.

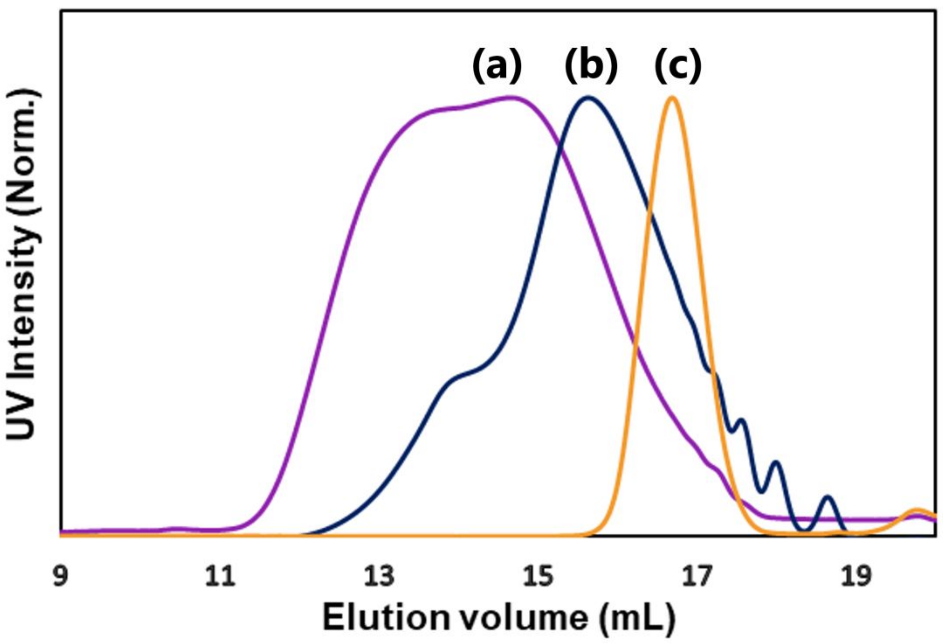

Figure S4. SEC UV traces of polymers: (a) tAB-1, (b) intermediate PNDI2T, and (c) tPIB-Br.

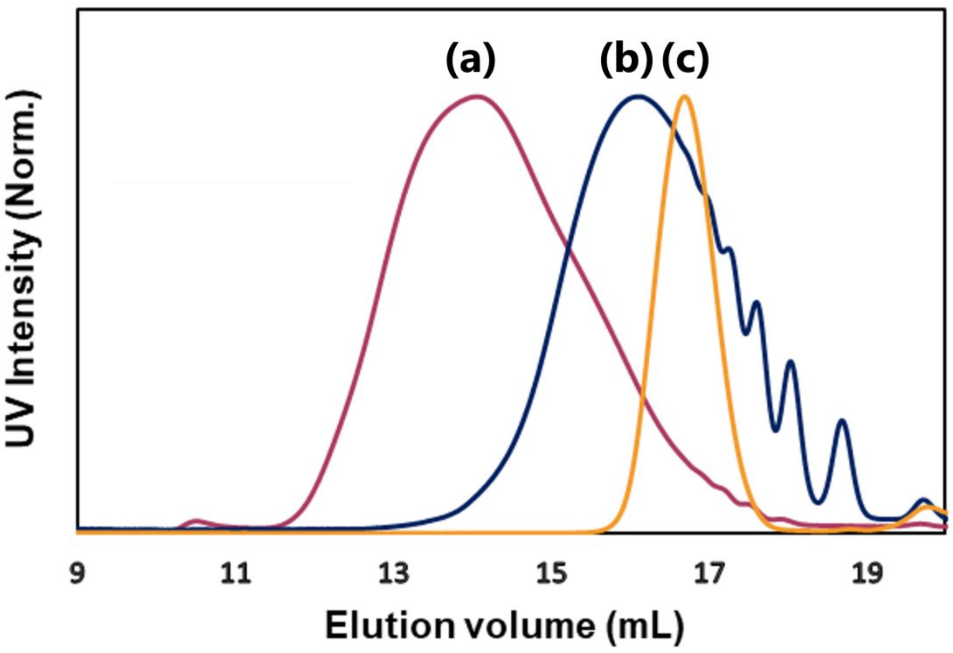

Figure S5. SEC UV traces of polymers: (a) tAB-2, (b) intermediate PNDI2T, and (c) tPIB-Br.

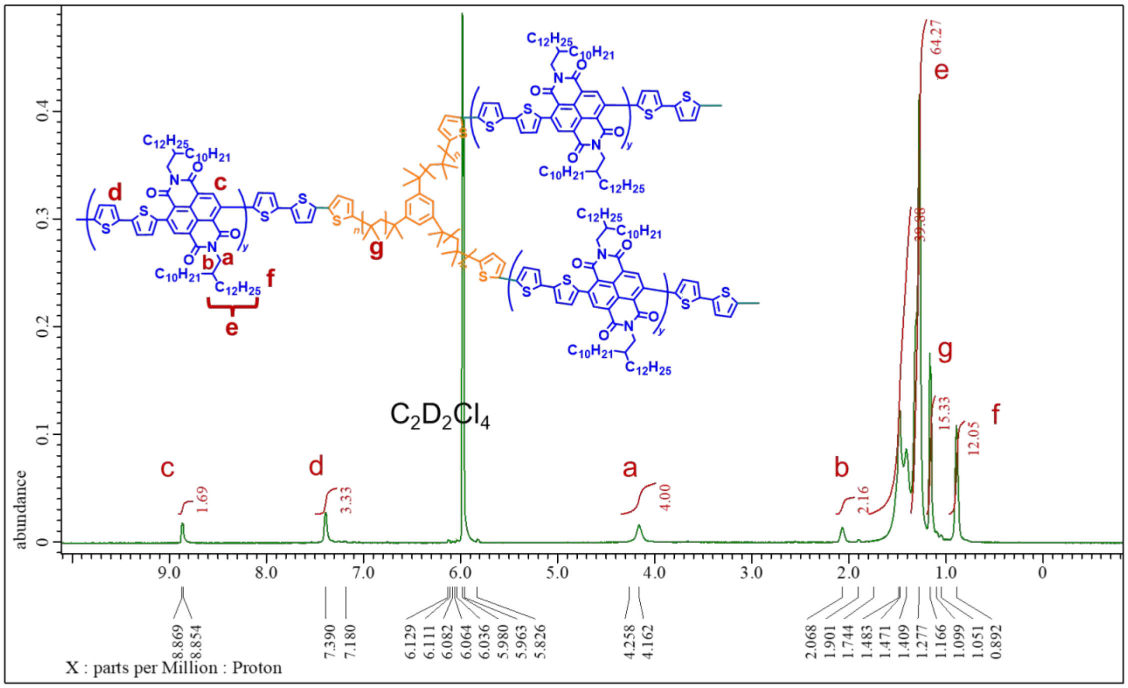

Figure S6. ^{1}H NMR spectra of tAB-1 in $C_2D_2Cl_4$.

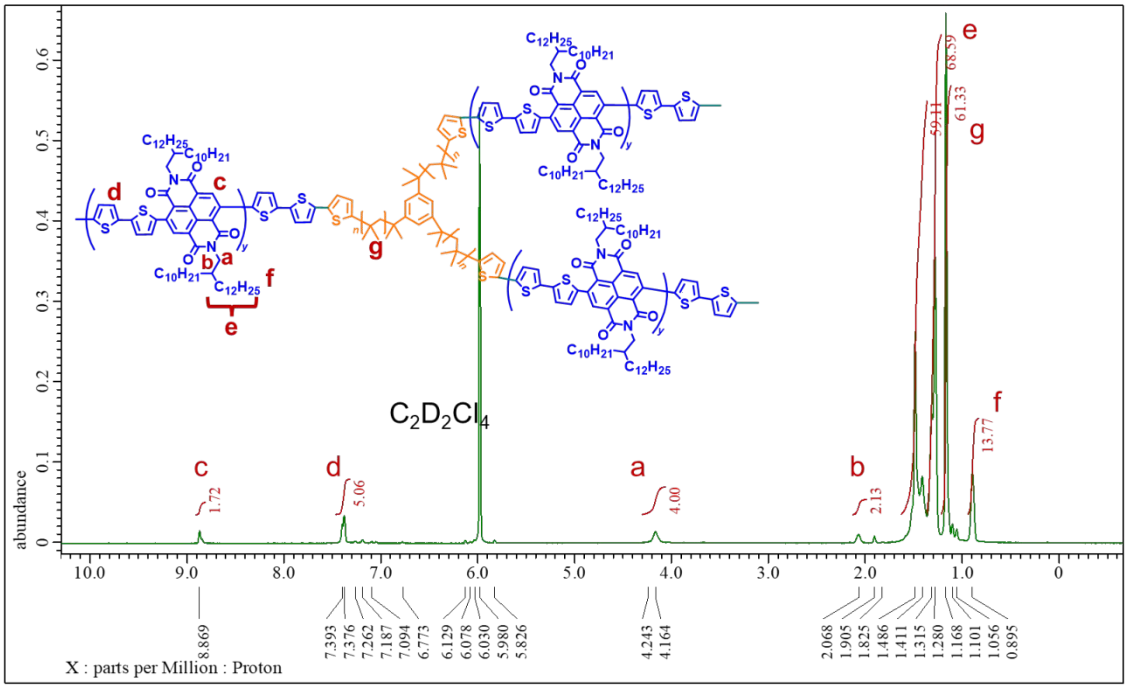

Figure S7. ^{1}H NMR spectra of tAB-2 in $C_2D_2Cl_4$.

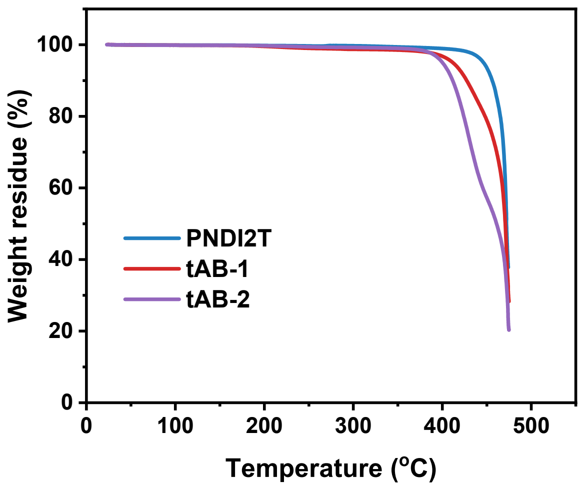

Figure S8. Thermogravimetric analysis (TGA) of the polymers studied.

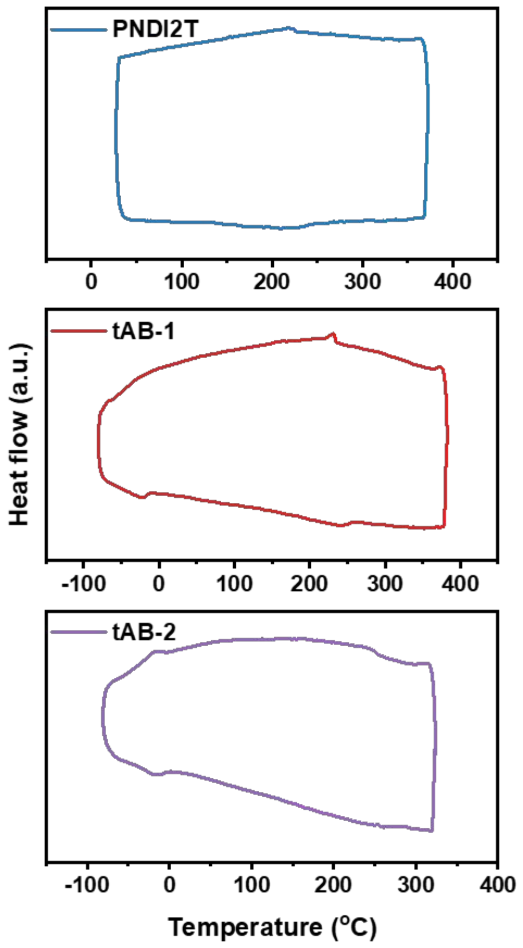

Figure S9. DSC thermograms of the polymers studied.

Table S1. Thermal properties of the studied polymers.

Polymer	TGA[a]	DSC[b]		
	$T_d^{5\%}$ (°C)	T_{m1} (°C)	T_{m2} (°C)	T_c (°C)
**tAB-1**	412	–40	240	230
**tAB-2**	400	–27	259	247

[a] Determined at a heating rate of 10 °C/min under a nitrogen flow. [b] Determined at ramping rate of 10 °C/min under a nitrogen flow. The 1st cooling and 2nd heating scans were taken.

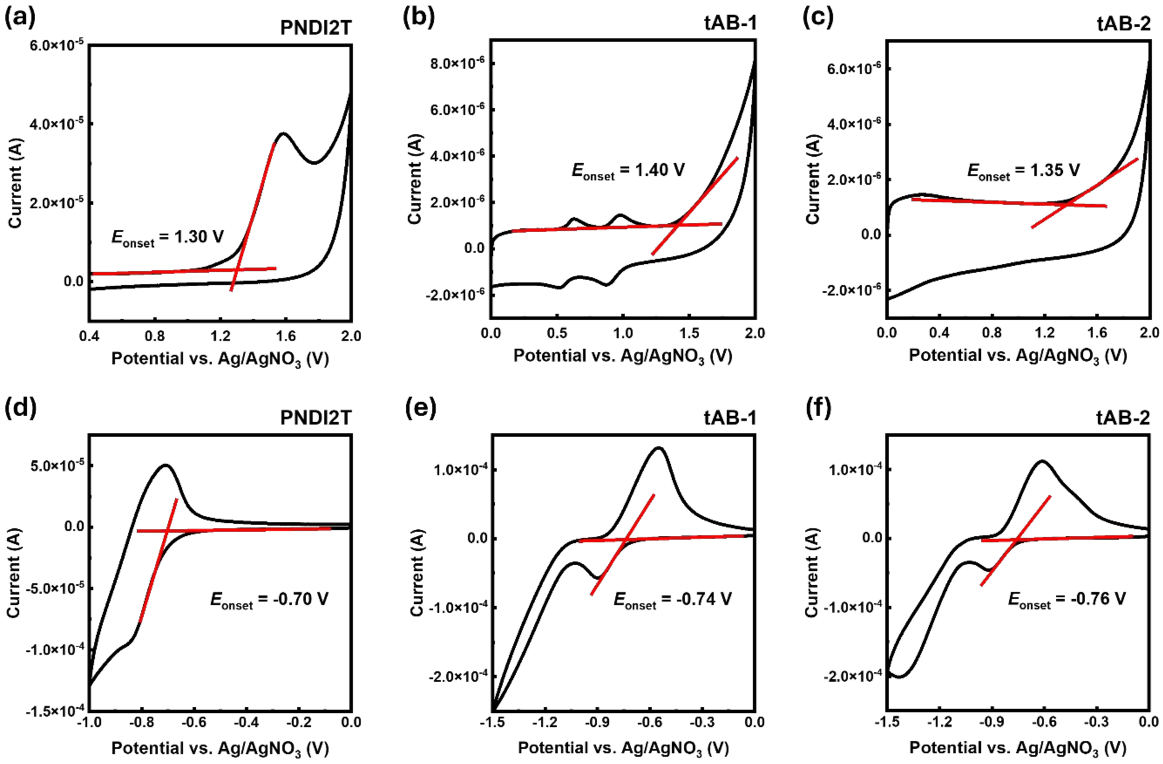

Figure S10. CV profiles of the polymer films coated on an ITO glass. Oxidation profiles of (a) PNDI2T, (b) tAB-1, and (c) tAB-2. Reduction profiles of (d) PNDI2T, (e) tAB-1, and (f) tAB-2.

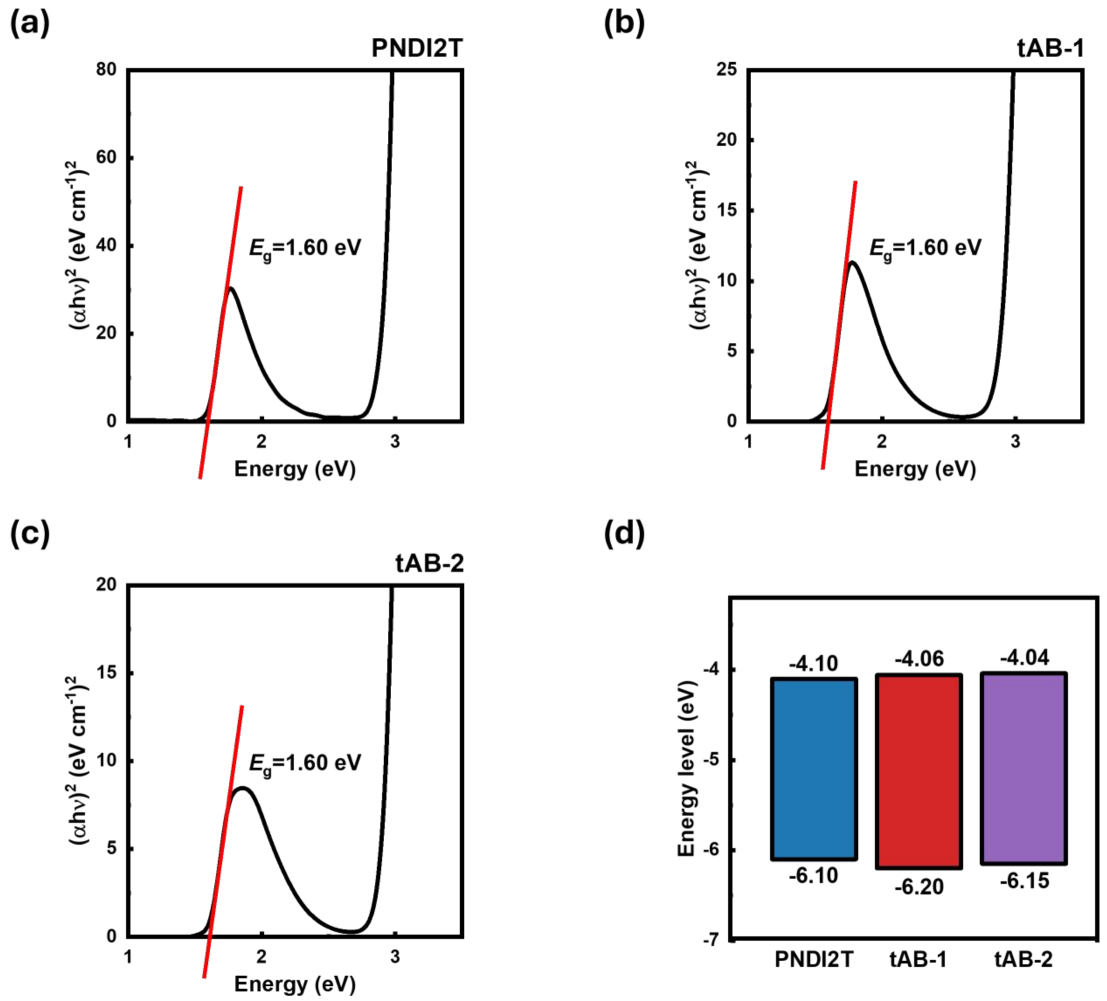

Figure S11. Tauc plot of studied polymer thin films: (a) PNDI2T, (b) tAB-1, and (c) tAB-2. (d) The energy level of the polymers studied was calculated from the CV.

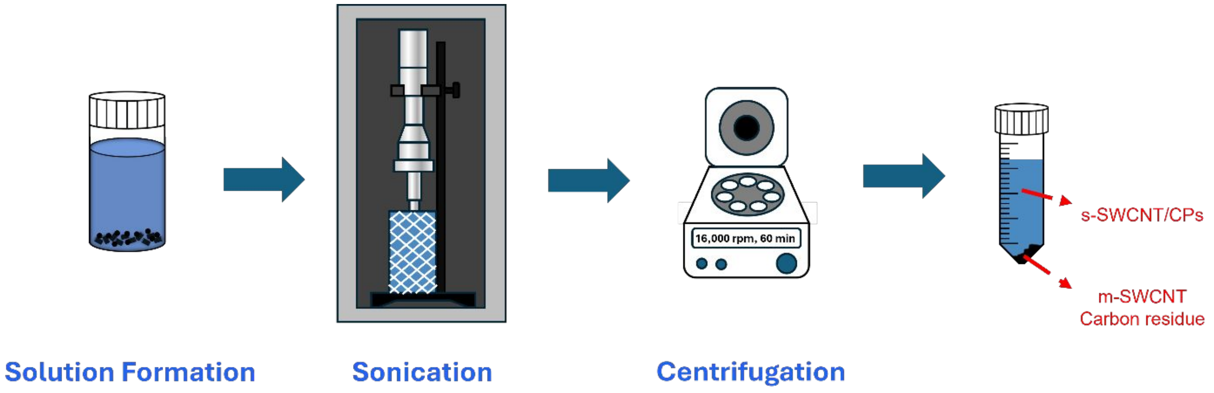

Figure S12. Preparation of the polymer/s-SWCNT composite, where CP: conjugated polymers, s-SWCNT: semiconducting SWCNT, and m-SWCNT: metallic SWCNT.

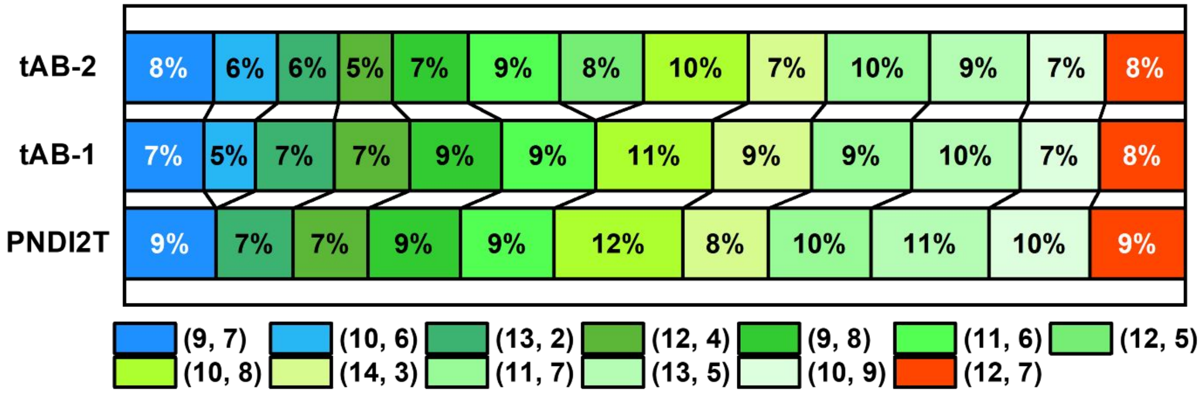

Figure S13. The chirality composition of the polymer/s-SWCNTs materials.

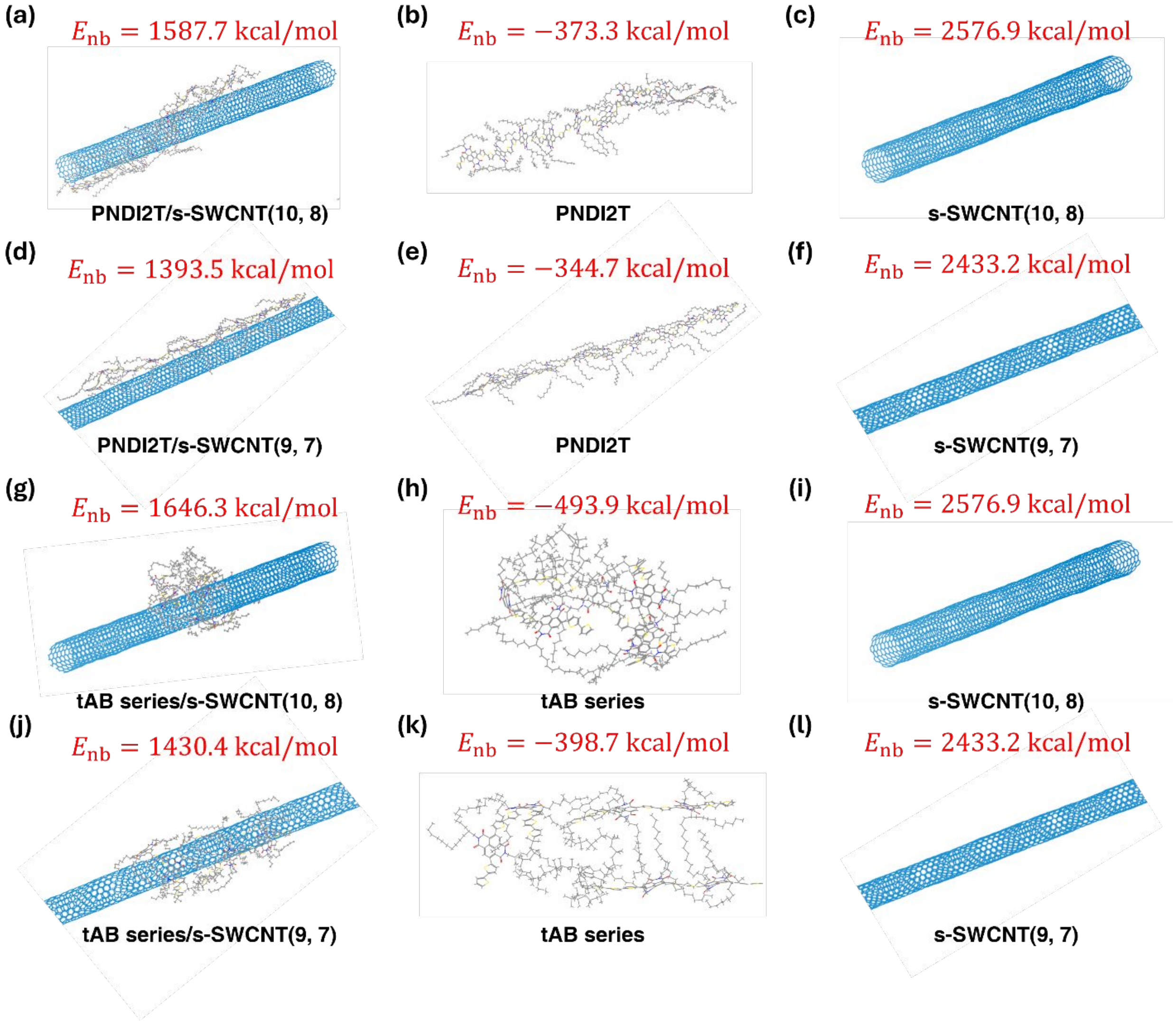

Figure S14. (a-f) The non-bonded energy and the stable structure of the (a,d) PNDI2T/s-SWCNT, (b,e) PNDI2T polymer, and (c,f) s-SWCNT in MD simulation. (g-l) The non-bonded energy and the stable structure of the (g,j) tAB series/s-SWCNT, (h,k) tAB series polymer, and (i,l) s-SWCNT in MD simulation. The s-SWCNT chiralities are (10, 8) for (a–c) and (g–i) and (9, 7) for (d–f) and (j–l).

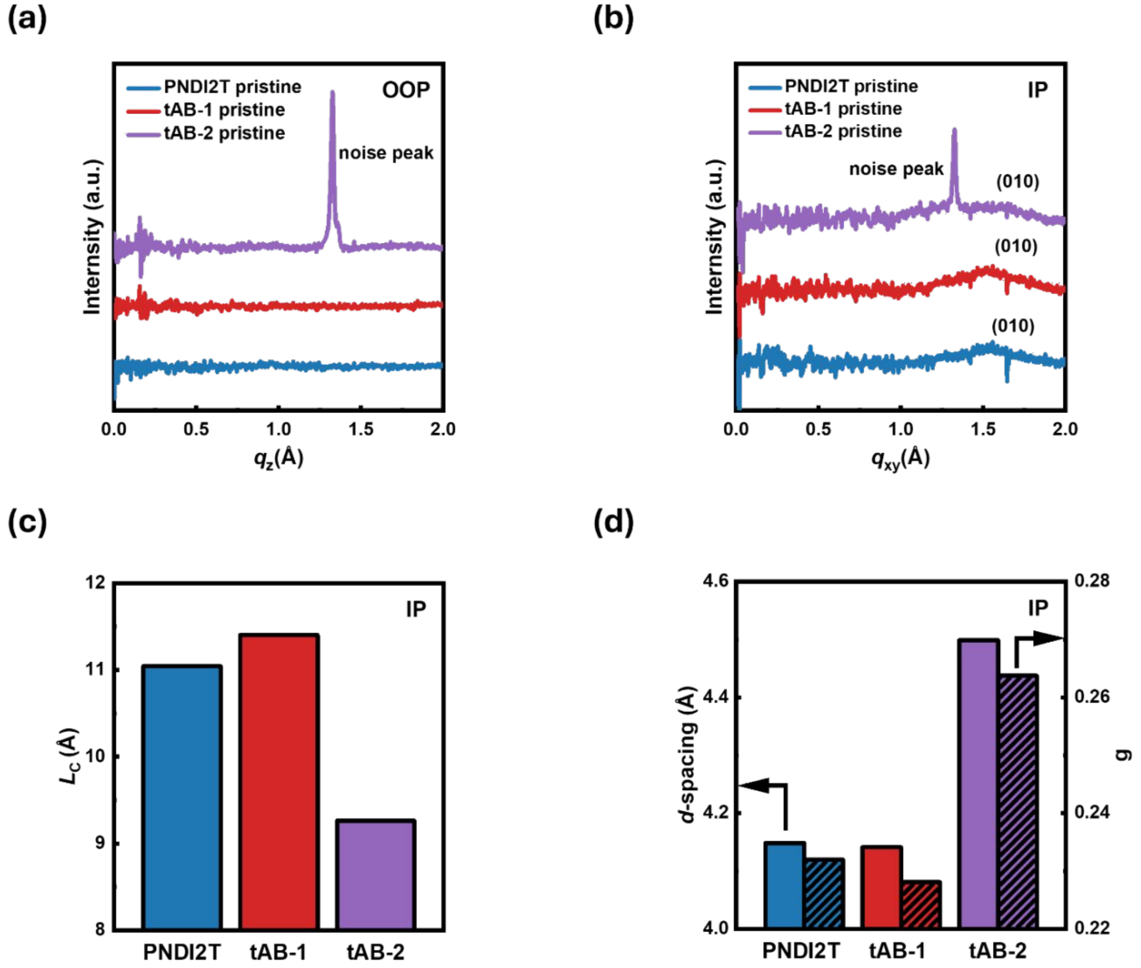

Figure S15. 1D GIWAXS of the polymer/s-SWCNT composite in (a) out-of-plane (OOP) direction and (b) in-plane (IP) direction. 1D GIWAXS parameter of the (010) signal in IP direction: (c) Coherence length (L_c) and (d) *d*-spacing of the IP (010) diffraction.

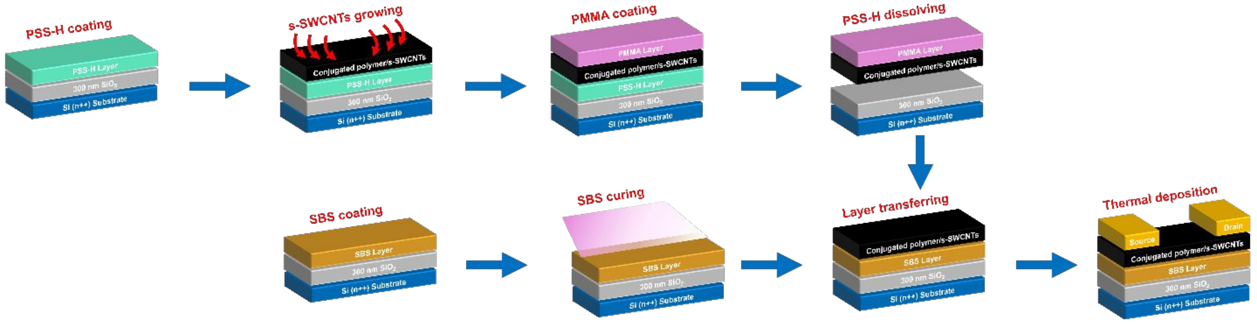

Figure S16. Fabrication of the photomemory devices based on the polymer/s-SWCNT composite thin film: physical adsorption, film transfer, printing, and thermal deposition of the source/drain electrodes onto the polymer/s-SWCNT thin film.

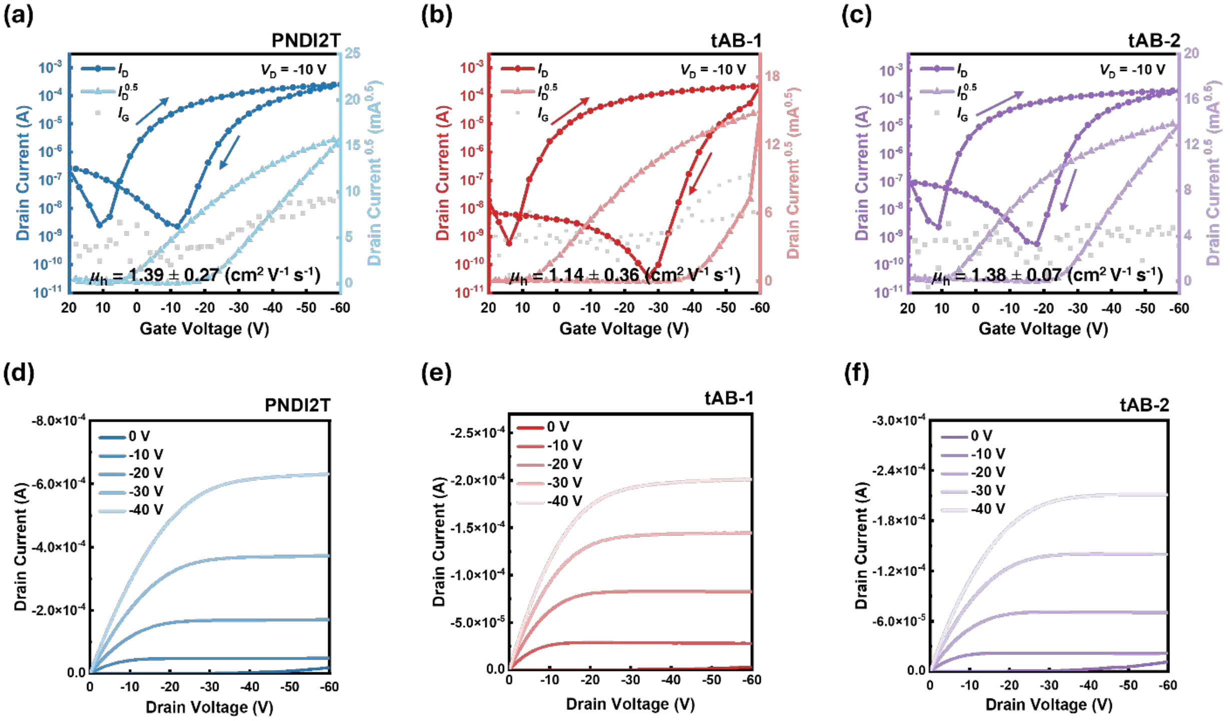

Figure S17. (a-c) Transfer curves and (d-f) output curves operate under $V_D = -10$ V for FET devices comprising (a,d) PNDI2T, (b,e) tAB-1, and (c,f) tAB-2. The sweeping rate is 16 V/s, and the transfer curve ranges from 20 to –60 V.

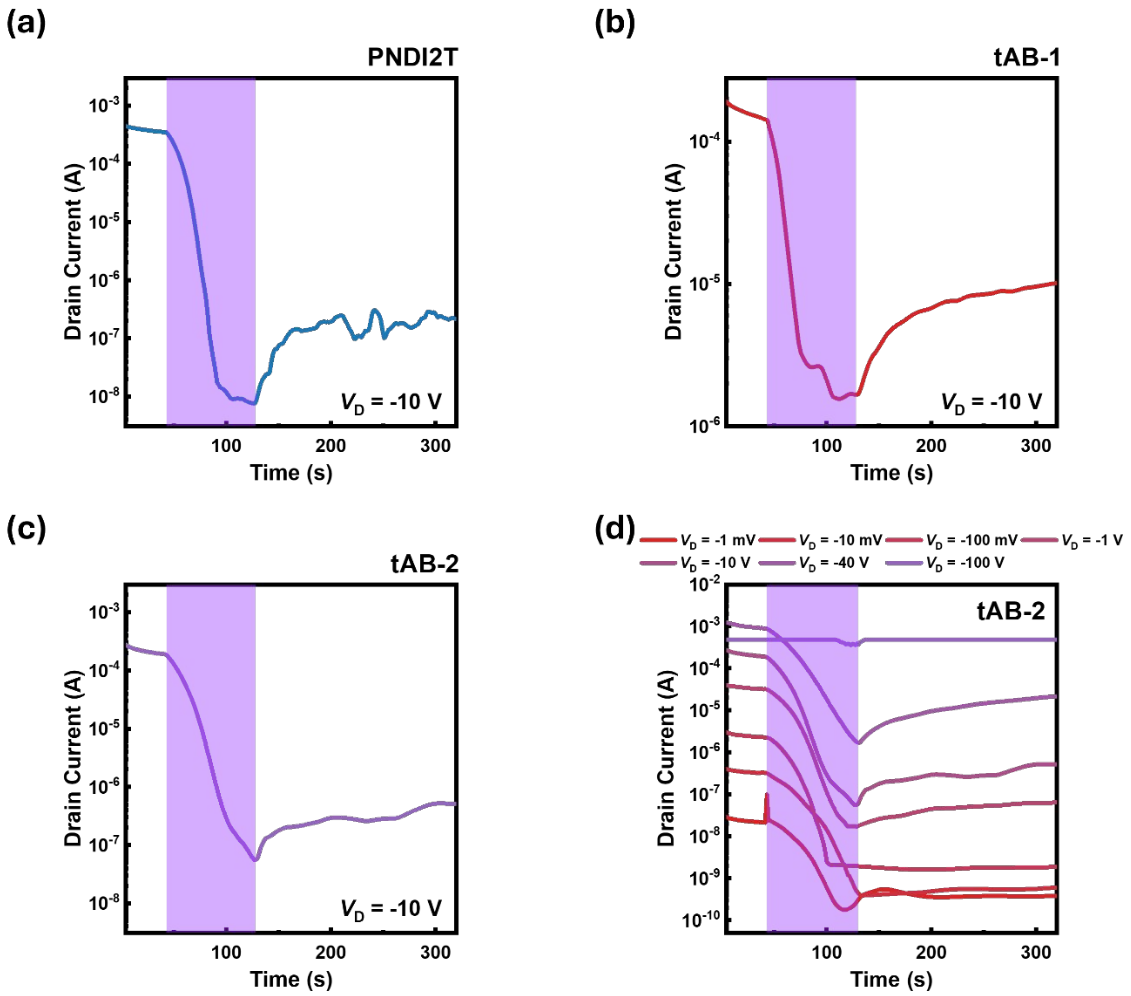

Figure S18. Transient memory characteristics of (a) PNDI2T/s-SWCNTs, (b) tAB-1/s-SWCNTs, and (c) tAB-2/s-SWCNTs: Optical erasing (OE) operation using 254-nm UV light for 90 s after a V_g = 100 V electrical writing (EW) pulse for 1 second. (d) Optical erasing (OE) operation using 254-nm UV light for 90 s under different drain voltages (V_D) operated in the tAB-2/s-SWCNTs device.
